# Macrophage Jak2 deficiency accelerates atherosclerosis through defects in cholesterol efflux

**DOI:** 10.1038/s42003-022-03078-5

**Published:** 2022-02-15

**Authors:** Idit Dotan, Jiaqi Yang, Jiro Ikeda, Ziv Roth, Evan Pollock-Tahiri, Harsh Desai, Tharini Sivasubramaniyam, Sonia Rehal, Josh Rapps, Yu Zhe Li, Helen Le, Gedaliah Farber, Edouard Alchami, Changting Xiao, Saraf Karim, Marcela Gronda, Michael F. Saikali, Amit Tirosh, Kay-Uwe Wagner, Jacques Genest, Aaron D. Schimmer, Vikas Gupta, Mark D. Minden, Carolyn L. Cummins, Gary F. Lewis, Clinton Robbins, Jenny Jongstra-Bilen, Myron Cybulsky, Minna Woo

**Affiliations:** 1 Toronto General Hospital Research Institute, University Health Network, Toronto, Canada; 2 Institute of Endocrinology, Beilinson Campus, Rabin Medical Center, Petach Tikva, Israel; 3 Program in Cell Biology, Peter Gilgan Centre for Research and Learning, Hospital for Sick Children, Toronto, Canada; 4 Department of Immunology, University of Toronto, Toronto, Canada; 5 Princess Margaret Cancer Centre, University Health Network, Toronto, Canada; 6 Department of Pharmaceutical Sciences, University of Toronto, Toronto, Canada; 7 Endocrine Cancer Genomics Center, Sheba Medical Center, Tel Hashomer, Israel; 8 Department of Oncology, Wayne State University School of Medicine and Tumor Biology Program, Barbara Ann Karmanos Cancer Institute, Detroit, MI USA; 9 Research Institute of the McGill University Health Centre, Royal Victoria Hospital, Montreal, QC Canada; 10 Division of Endocrinology and Metabolism, Department of Medicine, University Health Network and Sinai Health System, University of Toronto, Toronto, Canada

**Keywords:** Atherosclerosis, Experimental models of disease, Molecular medicine

## Abstract

Atherosclerosis is a chronic inflammatory condition in which macrophages play a major role. Janus kinase 2 (JAK2) is a pivotal molecule in inflammatory and metabolic signaling, and *Jak2*^*V617F*^ activating mutation has recently been implicated with enhancing clonal hematopoiesis and atherosclerosis. To determine the essential in vivo role of macrophage (M)-Jak2 in atherosclerosis, we generate atherosclerosis-prone ApoE-null mice deficient in M-Jak2. Contrary to our expectation, these mice exhibit increased plaque burden with no differences in macrophage proliferation, recruitment or bone marrow clonal expansion. Notably, M-Jak2-deficient bone marrow derived macrophages show a significant defect in cholesterol efflux. Pharmacologic JAK2 inhibition with ruxolitinib also leads to defects in cholesterol efflux and accelerates atherosclerosis. Liver X receptor agonist abolishes the efflux defect and attenuates the accelerated atherosclerosis that occurs with M-Jak2 deficiency. Macrophages of individuals with the *Jak2*^*V617F*^ mutation show increased efflux which is normalized when treated with a JAK2 inhibitor. Together, M-Jak2-deficiency leads to accelerated atherosclerosis primarily through defects in cholesterol efflux from macrophages.

## Abstract (web summary)

Loss of myeloid JAK2 promotes atherosclerosis in an ApoE knockout mouse model. This is associated with decreased cholesterol efflux from bone-marrow-derived macrophages.

## Introduction

Atherosclerosis is a complex chronic metabolic and inflammatory disease^1^. Atherosclerotic plaques develop upon recruitment of inflammatory cells, with lipid uptake leading to further aggravation of inflammation and plaque expansion. Endothelial dysfunction can initiate plaque development by exposing adhesion molecules such as intercellular adhesion molecule 1 (ICAM1) and vascular cell adhesion molecule 1 (VCAM1)^2^. Blood leukocytes, mainly monocytes but also lymphocytes, adhere to the activated endothelium and are recruited into the subendothelium^2^. There, monocytes differentiate into macrophages that proliferate, take up cholesterol and secrete inflammatory mediators. As disease progresses, smooth muscle cells (SMCs) also migrate into the plaque and secrete collagen and proteoglycans. Eventually, plaque macrophages and SMCs die and are cleared by other macrophages via efferocytosis^3^.

Plaque development is a dynamic process with opposing mechanisms such as cholesterol efflux, which can attenuate progression and even lead to regression^4,5^. This process has been shown to be clinically important in decreasing cardiovascular events^5,6^. Cholesterol efflux from the arterial wall serves as the first step of reverse cholesterol transport from the periphery into the liver. Lipid-poor apolipoprotein A1 (ApoA1) can initiate this process by binding to ATP-binding-cassette A1 (ABCA1) on foamy macrophage membrane to efflux cholesterol, leading to growth and maturation of high-density lipoprotein (HDL). HDL can also bind to ATP-binding cassette G1 (ABCG1), scavenger receptor B1 (SRB1), and efflux cholesterol via these transporters. Cholesterol can in turn be transferred to ApoB containing lipoproteins or directly to the liver via SRB1.

The Janus kinase-signal transducer and activator of transcription (JAK-STAT) is a major intracellular signal transduction pathway that mediates inflammatory and metabolic cues. JAK2, an important member in the JAK family, is expressed ubiquitously. It is the downstream mediator of a number of cytokine–receptor interactions, including growth hormone, erythropoietin, leptin, and IL6, and is activated through the binding of these ligands to their respective cell surface receptors. Upon ligand binding, JAK2, a non-receptor kinase, is recruited to and phosphorylates the intracellular-cytosolic domain of the receptor. This phosphorylation signals the recruitment and binding of specific STAT proteins, which in turn are phosphorylated, and dimerization leads to nuclear localization to regulate gene expression^7^. This complex signaling cascade is highly dependent on the cell type and the context in which JAK2 is activated, leading to a multitude of biological effects in cellular functions affecting growth, inflammation, and metabolism. For example, in vitro studies in a macrophage cell line showed a protective role of JAK2 against inflammatory responses^8^. Moreover, vagal stimulation of macrophages was shown to attenuate inflammation via the activation of the JAK2-STAT3 pathway^9^. We have shown on the other hand, that macrophage *Jak2* deficiency attenuates systemic inflammation in mice on a high-fat-diet^10^. Moreover, the activating *Jak2*^*V617F*^ mutation implicated in clonal hematopoiesis is associated with increased cardiovascular disease, and transgenic mice with this mutation showed accelerated atherosclerosis^11^. However, the essential in vivo role of myeloid *Jak2* (M-Jak2) and the specific mechanisms through which it can regulate atherosclerosis is unknown.

In this study, we show that *Apoe*^−*/−*^ mice deficient in M-Jak2 develop accelerated atherosclerosis compared to *Apoe*^*−/*−^ WT for M-Jak2. This was neither associated with obesity, impaired glucose homeostasis nor an increase in ApoB containing lipoproteins, but was associated with decreased cholesterol efflux from M-Jak2 bone marrow-derived macrophages (BMDM).

## Results

### M-Jak2 KO mice have accelerated atherosclerosis

In order to assess the essential role of Jak2 specifically in macrophages in vivo, we generated ApoE-null myeloid Jak2-specific knockout mice using the cre*LoxP* system under the control of the LysM promoter. ApoE-null LysMcre^+^-*Jak2*^*fl/fl*^ (denoted herein as M-Jak2 KO) were assessed and ApoE-null LysMcre^*+*^*-Jak2*^*WT/WT*^ (denoted herein as M-Jak2 WT) littermates served as controls. BMDM from M-Jak2 KO mice showed an efficient over 80% decrease in *Jak2* mRNA levels (*P* < 0.0001) and substantial reduction in JAK2 protein levels (Supplementary Fig. S1a–c). These results are in line with our previous work, in which *Jak2* mRNA and protein levels in thioglycolate-elicited peritoneal macrophages were decreased to a similar extent in M-Jak2 KO mice compared with littermate controls without ApoE-null mutation^10^. Moreover, as shown previously in these mice with macrophage-specific KO of *Jak2*^10^, we found that JAK2 protein levels were not affected in the liver, white adipose tissue (WAT) or hypothalamus of M-Jak2 KO and WT mice (Supplementary Fig. S1b, c).

**Fig. 1:**
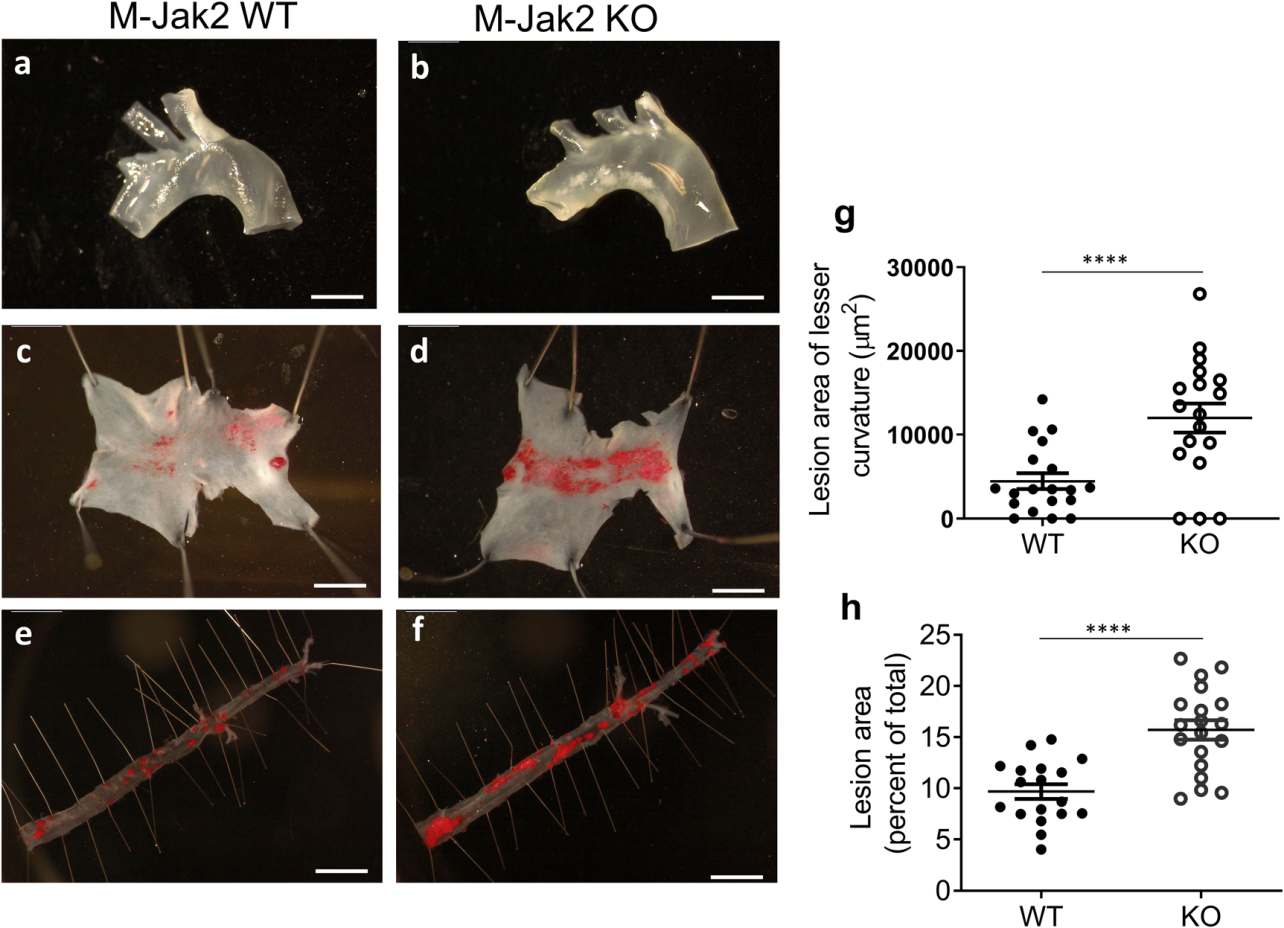
Accelerated atherosclerosis in M-Jak2*-*deficient mice. **a**, **b** Representative images of freshly isolated aortic arches isolated from M-Jak2 KO and WT control mice fed 3 weeks of HCD starting at 6 weeks of age. Scale bar, 5 mm. **c**, **d** Representative images of en face Oil-red-O staining of atherosclerotic plaques in the lesser curvature of the aortic arch after 3 weeks of HCD. Scale bar, 1 mm. **e**, **f** Representative images of en face Oil-red-O staining of atherosclerotic plaque area in the descending aorta after 16 weeks of HCD. Scale bar, 1 mm. **g** Quantification of atherosclerotic plaque area (mm^2^) in the aortic arch after 3 weeks of HCD (*n* = 18–19/genotype). **h** Quantification of atherosclerotic plaque area in the descending aorta after 16 weeks of HCD, expressed as a percentage of total area (*n* = 18–19/genotype). Statistical analysis: two-tailed unpaired *t* tests were performed. Data are presented as the mean ± SEM, ****P < 0.0001. See also Fig. 2, Supplementary Figs. S1, S2, S3, and S4.

**Fig. 2:**
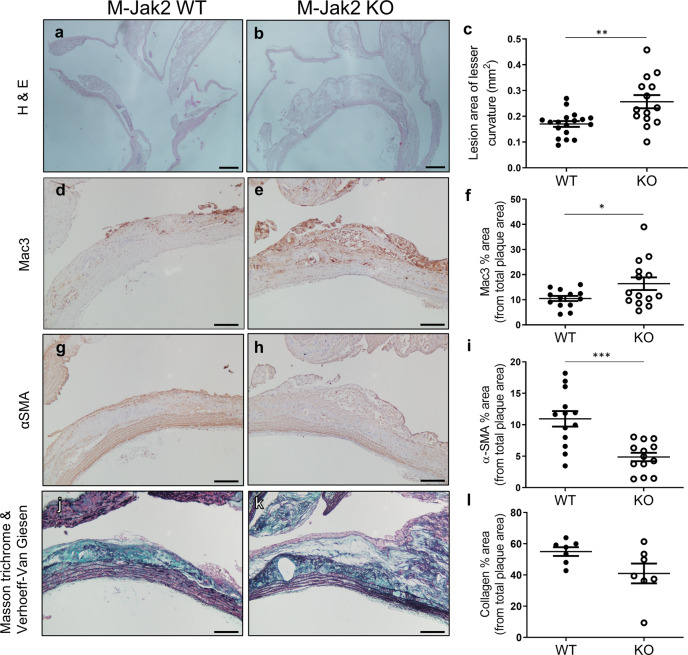
Plaque quantification and characterization in the lesser curvature of the aortic arch. **a**, **b** Representative images of the lesser curvature of the aortic arch sagittal sections stained with H&E from M-Jak2 KO and WT mice fed 16 weeks of HCD starting at 6 weeks of age. Scale bar, 200 µm. **c** Quantification of lesion size at the lesser curvature (mm^2^) (*n* = 14–18/genotype). **d**, **e** Representative images of the lesser curvature immunostained with anti-Mac3. Scale bar, 100 μm. **f** Quantification of Mac3 immunostained area expressed as a percentage of total plaque area (*n* = 13–14/genotype). **g**, **h** Representative images of the lesser curvature immunostained with anti-α-smooth muscle actin (SMA). Scale bar, 100 μm. **i** Quantification of α-SMA immunostained area expressed as a percentage of total plaque area (*n* = 13/genotype). **j**, **k** Representative images of the lesser curvature from aortic arch sections combined stained with Masson trichrome and Verhoeff Van Giesen. Scale bars, 100 μm. **l** Quantification of collagen expressed as a percentage of total plaque area using Masson trichrome stain (*n* = 7/genotype). Statistical analysis: two-tailed unpaired *t* tests were performed. Data are presented as the mean ± SEM, *P < 0.05, **P < 0.01, ***P < 0.001. See also Fig. 1 and Supplementary Fig. S4.

M-Jak2 KO and M-Jak2 WT littermates were fed an atherogenic high cholesterol diet (HCD) consisting of 21.2% fat and 1.5 g/kg cholesterol starting at 6 weeks of age. As early as 3 weeks on HCD, aortic arches of M-Jak2 KO mice showed an increase in plaque burden compared to WT controls by gross appearance (Fig. 1a, b). Quantification of en face plaque burden of aortic arches by Oil-red-O staining showed over 2.5-fold increase in M-Jak2 KO mice compared to WT littermate controls (Fig. 1c, d, g). This increase in atherosclerotic plaque burden persisted to 16 weeks on HCD, whereby the descending aorta, aortic arch, and aortic sinus showed increased plaque content (Figs. 1e, f, h and 2a–c, and Supplementary Fig. S2). Similar results were found in females in which M-*Jak2* KO mice accumulated more lipid-rich plaque burden in the aortic arch and the descending aorta compared to WT control female mice (Supplementary Fig. S3). Further characterization revealed that plaques from M-Jak2 KO mice had larger necrotic cores (Supplementary Fig. S4a–c), as well as increased Mac3-positive area and percentage of total plaque area (Fig. 2d–f and Supplementary Fig. S4d). Moreover, M-Jak2 KO mice had decreased smooth muscle actin (SMA) positive area and percentage of total plaque area in their lesser curvature of aortic arches (Fig. 2g–i and Supplementary Fig. S4e), in keeping with more advanced plaque progression that is associated with loss of SMA that maintains plaque stability. Collagen and elastic fiber contents were similar (Fig. 2j–l and Supplementary Fig. S4f–i). Overall, M-Jak2 KO mice show increased atherosclerotic burden, with features of advanced plaque progression as shown by larger necrotic cores, and higher macrophage and lower smooth muscle cell content.

### M-Jak2 KO mice do not exhibit adverse metabolic parameters

To understand the potential systemic metabolic abnormalities that may contribute to advanced atherosclerosis seen in M-Jak2 KO mice, we assessed various parameters including body weight, cholesterol levels, and glucose homeostasis. M-Jak2 KO mice had less weight gain throughout the period of 16 weeks on HCD in comparison to their WT controls (Supplementary Fig. S5a). Lipid profile after 16 weeks on HCD, including total cholesterol, low-density lipoprotein (LDL), high-density lipoprotein (HDL), triglycerides (TG), and non-HDL cholesterol, was similar between M-Jak2 KO and WT mice (Supplementary Fig. S5b–f). Fasting blood glucose levels were also similar at baseline (Supplementary Fig. S5g) and lower after 16 weeks on HCD for the M-Jak2 KO mice (Supplementary Fig. S5j). Glucose-tolerance test (GTT) and insulin-tolerance test (ITT) were similar at baseline (Supplementary Fig. S5h, i), but after 16 weeks on HCD, M-Jak2 KO mice had improved glucose tolerance and more glucose lowering in response to insulin compared to M-Jak2 WT mice (Supplementary Fig. S5k, l). Overall, these changes in metabolic parameters were similar to those observed in M-Jak2 KO mice without the ApoE-null mutation^10^, and would likely not contribute to the increased atherosclerotic burden seen in the M-Jak2 KO mice.

### Macrophage recruitment and proliferation within atherosclerotic plaque

Macrophages play a pivotal role in atherogenesis by several mechanisms. Circulating monocytes are actively recruited to and differentiate into macrophages within the plaque, where they can proliferate, take up cholesterol and become foam cells, thereby leading to plaque expansion^12^. We assessed macrophage recruitment to the plaques by measuring 24-h BrdU incorporation of CD45^+^ cells within lesions in the lesser curvature of the aortic arch. BrdU^+^CD45^+^-stained surfaces were similar between M-Jak2 KO and WT controls (Supplementary Fig. S6a–i). Macrophage proliferation within plaques was assessed using a 3-h BrdU incorporation, which showed similar BrdU^+^CD68^+^-stained surfaces between WT and KO groups (Supplementary Fig. S6j–r). Together, these results show that monocyte recruitment and macrophage proliferation within plaques were not affected by *Jak2*-deficiency in myeloid cells.

### Macrophage Jak2 in inflammatory response

JAK2 is generally known to mediate inflammatory response to many cytokines. Since atherogenesis is driven by a chronic inflammatory process, we assessed whether Jak2 deficiency enhances the inflammatory state of BMDM at baseline or their response to lipopolysaccharide (LPS) by comparing the mRNA levels of the pro-inflammatory genes *IL1β, IL6*, and *CCL5* between M-Jak2 KO and WT mice. In parallel, we interrogated the inflammatory effects of lipid loading in cells pre-loaded with oxidized low-density lipoproteins (oxLDL). Pro-inflammatory gene expression was similar between BMDM of M-Jak2 KO and WT mice under basal or LPS-stimulated conditions (Supplementary Fig. S7a–c). OxLDL loading did not significantly change the basal expression of these genes in macrophages of both genotypes. A significant reduction in LPS-induced expression of *IL1β*, *IL6*, and *CCL5* was observed in oxLDL-loaded M-Jak2 WT BMDM as shown by others^13,14^, and *Jak2* deficiency did not alter the negative regulatory role of oxLDL on LPS-induced inflammatory gene expression (Supplementary Fig. S7a–c). Overall, this data show that *Jak2*-deficiency did not have any essential regulatory role in the inflammatory response of macrophages. In support of these findings, there was no difference in circulating IL1β and IL6 levels, nor other pro-inflammatory cytokines such as TNFα, MCP1, or IL12 in the serum of M-Jak2 WT and KO mice fed atherogenic diet for 3 or 16 weeks (Supplementary Fig. S7d–h). Interestingly, anti-inflammatory IL10 levels were decreased in M-Jak2 KO mice compared to M-Jak2 WT mice (Supplementary Fig. S7i). We also examined gene expression of M2-like markers, both basally and in response to oxLDL in BMDMs, and found no significant difference in mRNA level of *MRC1*, *ARG1, PPARG*, and *FIZZ1* between M-Jak2 KO and WT groups (Supplementary Fig. S7j–m).

Polarization experiments comparing M1 and M2 markers after stimulation with LPS and IL4 respectively showed similar *IL1β* and *IL6* but heightened induction of *Itgax* and *IFNα* in response to LPS in M-Jak2 KO compared to WT BMDM (Supplementary Fig. S8a–d). In response to IL4, M-Jak2 KO BMDM showed lower mRNA levels of *ARG1* and *MGL2*, with some similar mRNA (*FIZZ1* and *MGL1*) and higher *CHI313* levels compared to M-Jak2 WT BMDM (Supplementary Fig. S8e–j). These results show mixed polarization with some increased M1 and mixed M2 polarization. Moreover, circulating leukocytes from mice after 16 weeks of HCD did not show a significant difference between M-Jak2 KO and WT mice (Supplementary Fig. S9). Collectively, these results show that inflammation does not likely play a predominant role in the accelerated atherosclerosis observed in M-Jak2 KO mice.

### Jak2 regulates cholesterol efflux from lipid-laden macrophages

**Fig. 3:**
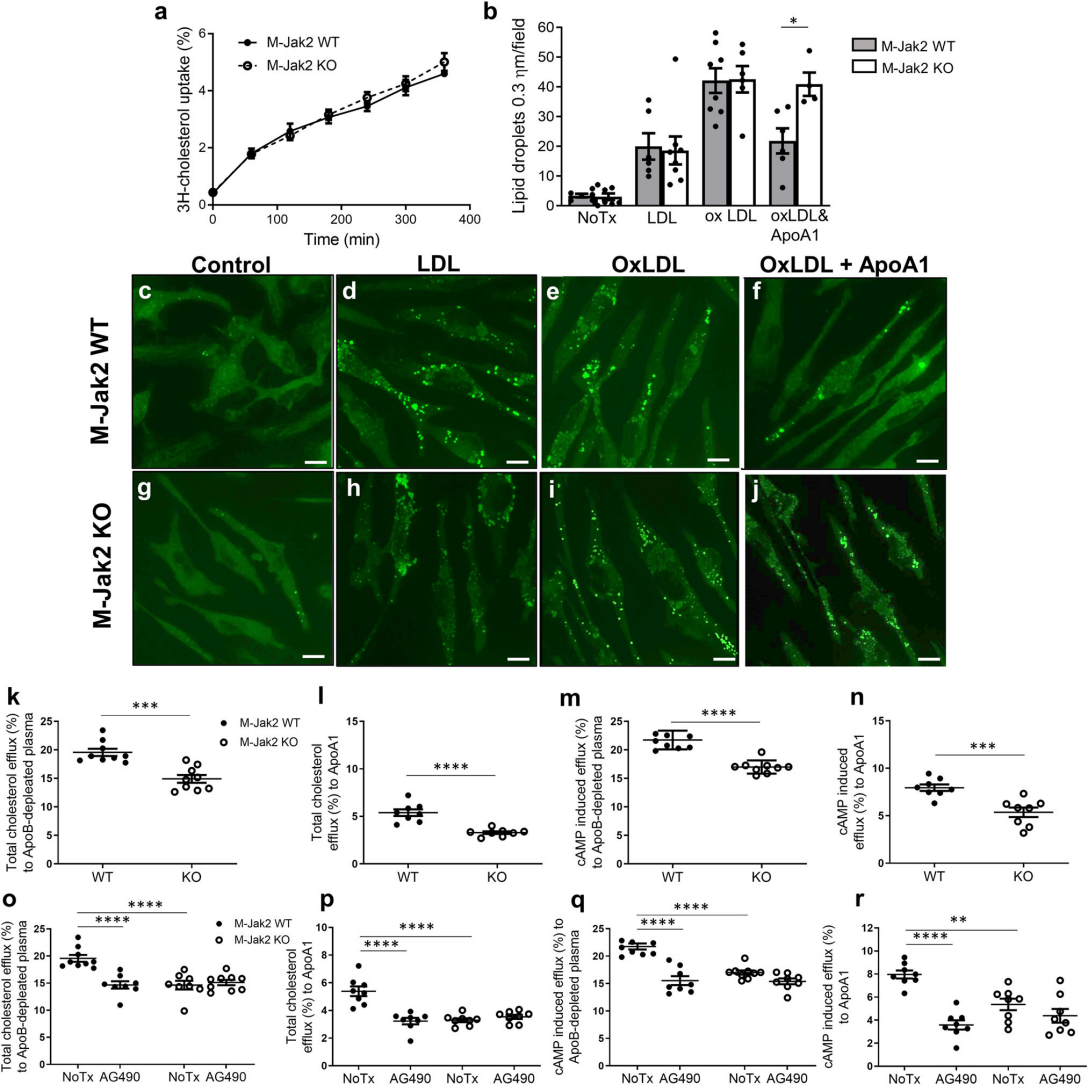
Decreased cholesterol efflux in *Jak2*-deficient macrophages. **a** Radioactive cholesterol uptake in BMDM of M-Jak2 KO and WT mice (*n* = 4–6/genotype). **b** Quantification of the number of lipid droplets sized 0.3 µm per field stained with BODIPY, in BMDM untreated or treated with LDL, oxLDL, or combined oxLDL and ApoA1 for 24 h (*n* = 4–8/genotype). **c**–**j** Representative images of BMDM in the conditions described in (**b**). Scale bar, 6.6 µm. **k**, **l** Cholesterol efflux measurements using radioactive cholesterol from BMDM; total unstimulated, in response to ApoB-depleted plasma (*n* = 9/group) and to ApoA1 (*n* = 8/group). **m**, **n** BMDM stimulated with cAMP to induce ABCA1, and total efflux from M-Jak2 KO and WT BMDM in response to ApoB-depleted plasma (*n* = 9/group) and to ApoA1 (*n* = 8/group). **o**–**r** Cholesterol efflux measurements after JAK2 inhibition with AG490 from M-Jak2 KO and WT BMDM (*n* = 8/group). Statistical analysis: **a**, **b** Multiple *t* tests with Holm–Sidak correction, **k**–**n** two-tailed unpaired *t* tests, and (**o**–**r**) two-way ANOVA with Tukey’s multiple comparisons were performed. Data are presented as the mean ± SEM, *P < 0.05, **P < 0.01, ***P < 0.001, ****P < 0.0001. See also Supplementary Figs. S10.

Jak2 has been implicated in cellular lipid homeostasis in vitro^15–17^; however, its role in vivo is unknown. Macrophage cholesterol content is the balance between their cholesterol uptake and efflux. To assess the effect of Jak2 in cholesterol uptake, we loaded BMDM from M-Jak2 KO and WT mice with ^3^H-cholesterol, and measured uptake for 6 h. We observed accumulating cholesterol over this time in both M-Jak2 KO and WT BMDM without any differences between the two groups (Fig. 3a). In order to compare cholesterol uptake at a steady state, we loaded BMDM with LDL or oxLDL for 24 h and stained with BODIPY to visualize lipid uptake. We observed a significant induction of lipid uptake in response to LDL with a further induction with oxLDL in both M-Jak2 KO and WT BMDM, with no differences between genotypes both basally and in response to lipid exposure (Fig. 3b–e, g–i). In line with similar lipid uptake between M-Jak2 KO and WT BMDM, mRNA levels of genes implicated in the uptake of various cholesterol particles, such as *LDLR*, CD*36*, *MSR1*, *MACRO*, *TLR4*, and *OLR1* also showed no differences between KO and WT BMDM (Supplementary Fig. S10).

JAK2 has been shown to enhance cholesterol efflux in vitro by altering ABCA1 function^15,16^. Under physiological conditions, cholesterol efflux is initiated by cholesterol being transported to ApoA1 via ABCA1, and as the particle develops into mature HDL, cholesterol transport occurs via ABCG1 and SRB1^18,19^. We therefore measured cholesterol efflux from BMDM to ApoA1, which showed a defect in M-Jak2 KO compared to WT BMDM as assessed by BODIPY staining (Fig. 3b, f, j).

We also assessed ^3^H-cholesterol efflux to ApoB-depleted plasma (containing ApoA1 and all sizes of HDL) and to purified ApoA1, to examine the initial transport via ABCA1. ^3^H-cholesterol efflux to ApoB-depleted plasma was decreased by 23.7% in M-Jak2 KO compared to WT BMDM (14.9% vs 19.6%; *P* = 0.0001; Fig. 3k). This defect was further accentuated when ^3^H-cholesterol efflux was measured to purified ApoA1, whereby Jak2-deficient BMDM had 37.9% lower efflux compared to WT controls (3.3% vs 5.3%; *P* < 0.0001, 37.9%; Fig. 3l). We next assessed whether this defect would persist after ABCA1 induction with cAMP. Similar to non-induced conditions, we observed a significant defect in cholesterol efflux in M-Jak2 KO BMDM compared to WT controls (17% vs. 21.74%, *P* < 0.0001, for ApoB-depleted plasma and 5.39% vs. 7.96%, *P* < 0.001 for ApoA1; Fig. 3m, n). Overall, M-Jak2 appears to play an essential regulatory role in cholesterol efflux, and induction with cAMP does not appear to overcome the cholesterol efflux defect seen with M-Jak2 deficiency.

**Fig. 4:**
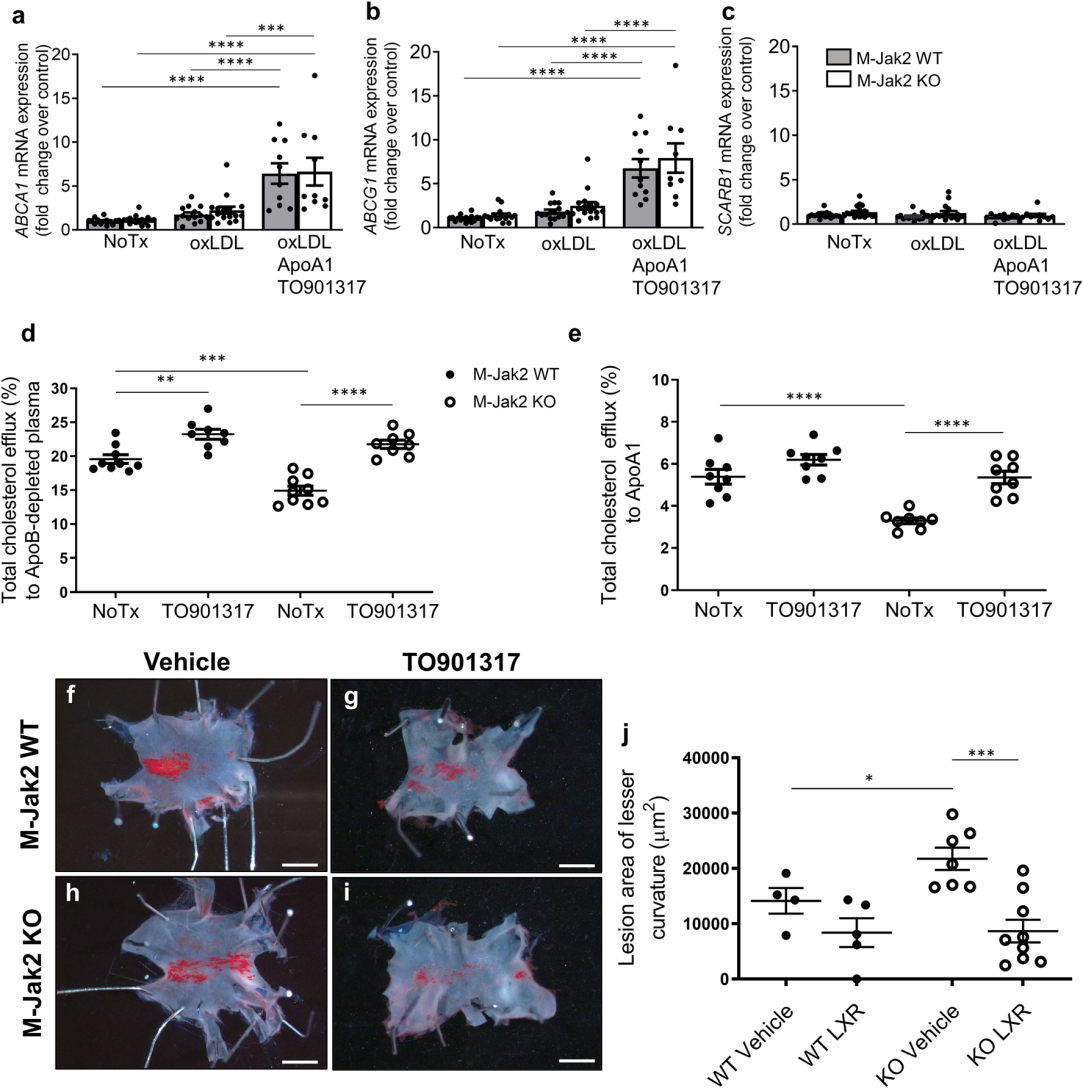
Cholesterol efflux from M-Jak2 KO WT BMDM with LXR agonist. **a**–**c** mRNA level of *ABCA1, ABCG1*, and *SCARB1* in BMDM under basal conditions, after loading with oxLDL or after loading with oxLDL and treating with TO901317 with ApoA1 to induce efflux (*n* = 10–19/group). **d**, **e** Cholesterol efflux from BMDM of M-Jak2 KO and WT mice treated with the LXR agonist, TO901317 in response to ApoB-depleted plasma (*n* = 8/group) and to purified ApoA1 (*n* = 8/group). **f**–**i** Representative en face images of Oil-red-O-stained lesser curvature of the aortic arch from M-Jak2 KO and WT mice treated with TO901317 or vehicle while on HCD for 3 weeks starting at 6 weeks of age. Scale bar, 1 mm (**j**) and their quantification (*n* = 4–9/group). Statistical analysis: **a**–**e** two-way ANOVA with Tukey’s multiple comparisons and (**j**) multiple *t* tests with Holm–Sidak correction were performed. Data are presented as the mean ± SEM, *P < 0.05, **P < 0.01, ***P < 0.001, ****P < 0.0001. See also Supplementary Fig. S11.

To test whether defects in cholesterol efflux observed with genetic Jak2 deficiency may also be affected by pharmacologic inhibition, we examined the effects of the JAK2 inhibitor AG490. Similar to genetic deletion of M-Jak2, AG490-treated BMDM from M-Jak2 WT mice showed significantly lower efflux both to ApoB-depleted plasma (14.7% vs 19.6%; *P* < 0.0001) and to ApoA1 (3.2% vs 5.4%; *P* < 0.0001; Fig. 3o, p). AG490 exposure to BMDM from M-Jak2 KO on the other hand, did not lead to a further reduction in efflux when compared to the control group without AG490 (15.1% vs 14.6% vs. for ApoB-depleted plasma and 3.3% vs. 3.5% for ApoA1, *P* = NS for both; Fig. 3o, p). cAMP induction led to a persistent defect in efflux from WT BMDM to ApoB-depleted plasma when treated with AG490 (decreased from 21.7% to 15.5%, *P* < 0.0001), whereas there was no significant further reduction in efflux by AG490 in M-Jak2 KO BMDM (from 17% to 15.4%; Fig. 3q, r). Efflux to ApoA1 after cAMP induction decreased from 8.0 to 3.6% (*P* < 0.0001) for WT BMDM treated with AG490, whereas no significant change was observed in KO BMDM (5.4% vs. 4.4%, Fig. 3q, r). Gene expression of *ABCA1, ABCG1*, and *SCARB1* showed no difference between M-Jak2 KO and WT BMDM basally and after 24-h treatment with OxLDL (Fig. 4a–c). Taken together, these results show that Jak2 deficiency leads to a defect in cholesterol efflux, without changes in transcriptional levels of the factors that play an important role in this process.

### LXR agonist can overcome the cholesterol efflux defect in M-Jak2 KO BMDM and attenuate accelerated atherosclerosis in M-Jak2 KO mice

To assess whether the defect in cholesterol efflux plays a causal role in the accelerated atherosclerosis in M-Jak2 mice, we treated BMDM from M-Jak2 WT and KO mice with an LXR agonist TO901317 which is known to enhance cholesterol efflux through transcriptional upregulation of the cholesterol transporters ABCA1 and ABCG1^20^. As shown previously^20^, treatment with TO901317 in vitro led to a significant, over sixfold, induction of both ABCA1 and ABCG1 in BMDM of WT mice with similar induction of these protein levels in the M-Jak2 KO mice in response to oxLDL and ApoA1 (Fig. 4a, b and Supplementary Fig. S11). SRB1, a gene not affected by LXR^21^, was unchanged in response to either OxLDL or ApoA1 in both WT and KO groups (Fig. 4c). We next assessed for functional effects of this agonist, which, as expected significantly increased cholesterol efflux to ApoB-depleted plasma in BMDM of WT mice (19.6% vs. 23.2%, *P* = 0.0029). Importantly, we observed an increase in cholesterol efflux in BMDM of M-Jak2 KO mice to a similar level as WT controls (14.9% vs. 21.8% for ApoB-depleted plasma and 3.3% vs. 5.4% for ApoA1, *P* < 0.0001), thereby abolishing the defect in efflux that was present in the M-Jak2 KO BMDM (Fig. 4d, e).

To further explore the potential causal mechanism of M-Jak2 as a critical regulator of cholesterol efflux in affecting atherogenesis, we administered TO901317 or vehicle by daily oral gavage to M-Jak2 KO and WT mice fed HCD for a period of 3 weeks, starting at 6 weeks of age. We found that plaque burden in the lesser curvature of the aortic arch of the vehicle-treated groups was increased in M-Jak2 KO mice compared to WT controls. In contrast, in the TO901317-treated group, there was a significant attenuation in atherosclerotic plaque burden in M-Jak2 KO mice to a similar level as that of WT mice, thereby abolishing the difference in disease progression (Fig. 4f, j). These results support that M-Jak2 plays a critical regulatory role in cholesterol efflux that protects against atherosclerosis progression.

### Systemic treatment with JAK2 inhibitor leads to accelerated atherosclerosis

**Fig. 5:**
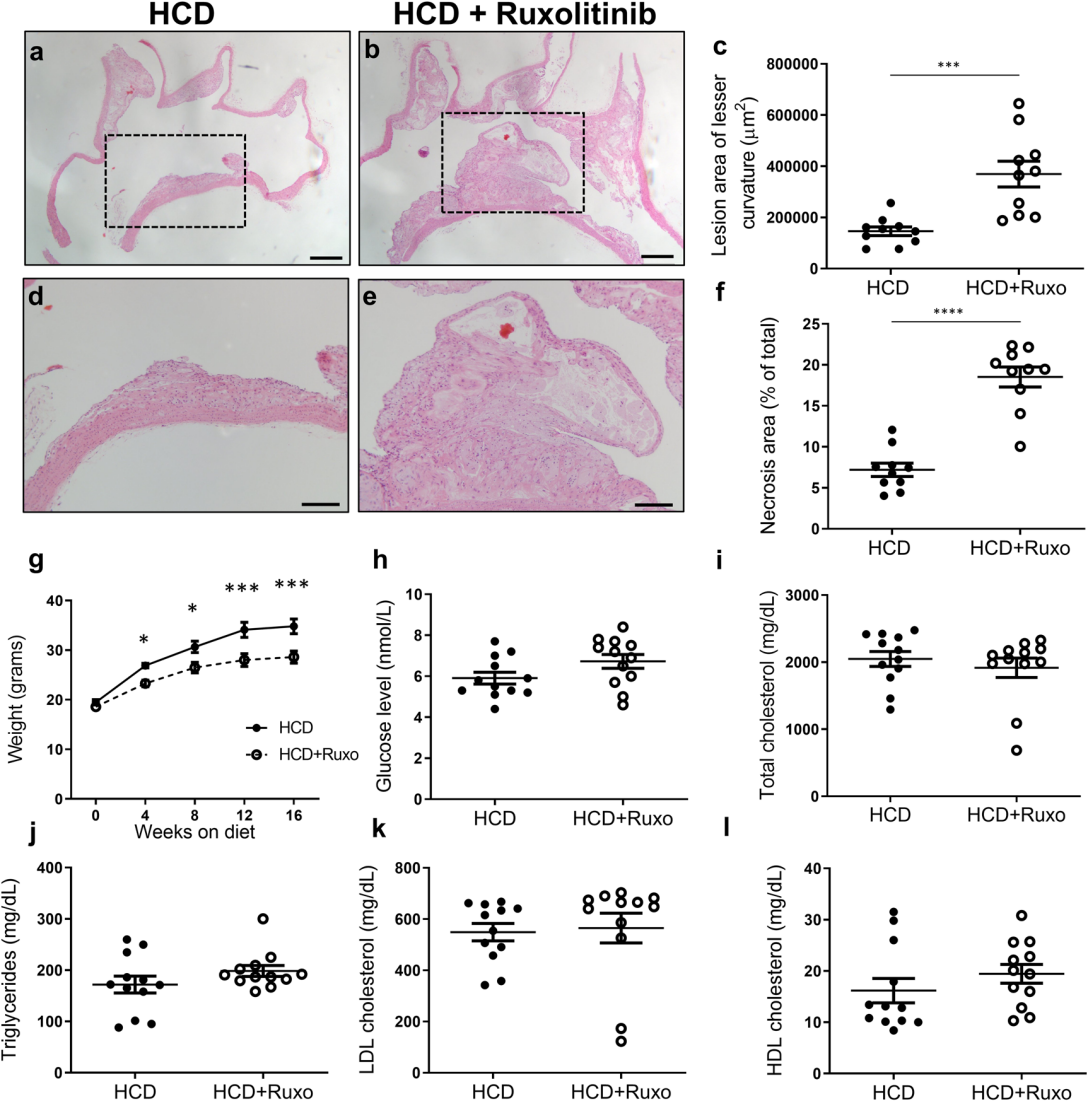
Effects of ruxolitinib in atherosclerosis. **a**, **b** Representative images of sagittal aortic arch sections stained with H&E from ApoE KO mice fed either HCD with or without ruxolitinib for 16 weeks. Scale bar, 200 µm. **c** Quantification of lesion size at the lesser curvature (µm^2^) (*n* = 10/group). **d**, **e** Magnification of region of interest from (**a**, **b**). Scale bar, 100 µm. **f** Quantification of the necrotic area within the plaque expressed as a percentage of total area (*n* = 10/group). **g** Body weights of ApoE KO mice treated with or without ruxolitinib while on 16 weeks of HCD (*n* = 12 per group). **h**–**l** and their fasting blood glucose and lipid levels (*n* = 12/group). Statistical analysis: **c**–**f**, **h**–**l**) two-tailed unpaired *t* tests and (**g**) multiple *t* tests with Holm–Sidak correction were performed. Data are presented as the mean ± SEM, *P < 0.05, ***P < 0.001, ****P < 0.0001. See also Supplementary Fig. S12.

JAK2 inhibitors, such as ruxolitinib, are currently approved for chronic autoimmune and hematologic diseases^22^. These are chronic conditions that require long-term therapy; and yet, the consequences of chronic exposure to such agents are not clear. In order to assess the effects of systemic exposure to JAK2 inhibitors on atherosclerosis, we fed ApoE-null mice with HCD with or without ruxolitinib for 16 weeks, starting at 6 weeks of age, and assessed for atherosclerotic burden. Following 16 weeks of ruxolitinib, the aortic arches showed a 2.5-fold increase in plaque burden (*P* = 0.0006) with larger necrotic cores compared to the control group without ruxolitinib (~7-fold higher, *P* < 0.0001, Fig. 5a–f). Interestingly, no difference in plaque burden was found in the descending aorta (Supplementary Fig. S12a–c). Furthermore, the ruxolitinib group had higher levels of serum IL6; however other cytokines were similar to the control group (Supplementary Fig. S12d–h). Also, lymphocytes were decreased in the ruxolitinib group; however, other blood parameters were similar to controls (Supplementary Fig. S12i–n). Furthermore, similar to M-Jak2 KO mice, ApoE-null mice on ruxolitinib had less weight gain compared to controls (Fig. 5g). In addition, fasting glucose and lipid profile at the end of the 16 weeks of treatment were not affected by ruxolitinib (Fig. 5h–l).

### Myeloid Jak2-deficiency does not affect clonal hematopoiesis

*Jak2*^*V617F*^ is an activating mutation that is associated with myeloproliferative disorders^23^. As well, this mutation in Jak2, along with several other genes implicated in clonal hematopoiesis, has recently been shown to be linked to increased risk of cardiovascular disease^24^. We therefore assessed whether myeloid Jak2 deletion may affect clonal hematopoiesis which may have contributed to the accelerated atherosclerosis seen in M-Jak2 KO mice. To this end, we performed clonogenic growth assay on freshly collected bone marrow from M-Jak2 KO and WT mice, and found no differences in the granulomonocytic colony forming units (Supplementary Fig. S13).

### Macrophages from humans harboring the *Jak2*^*V617F*^ mutation show increased cholesterol efflux

**Fig. 6:**
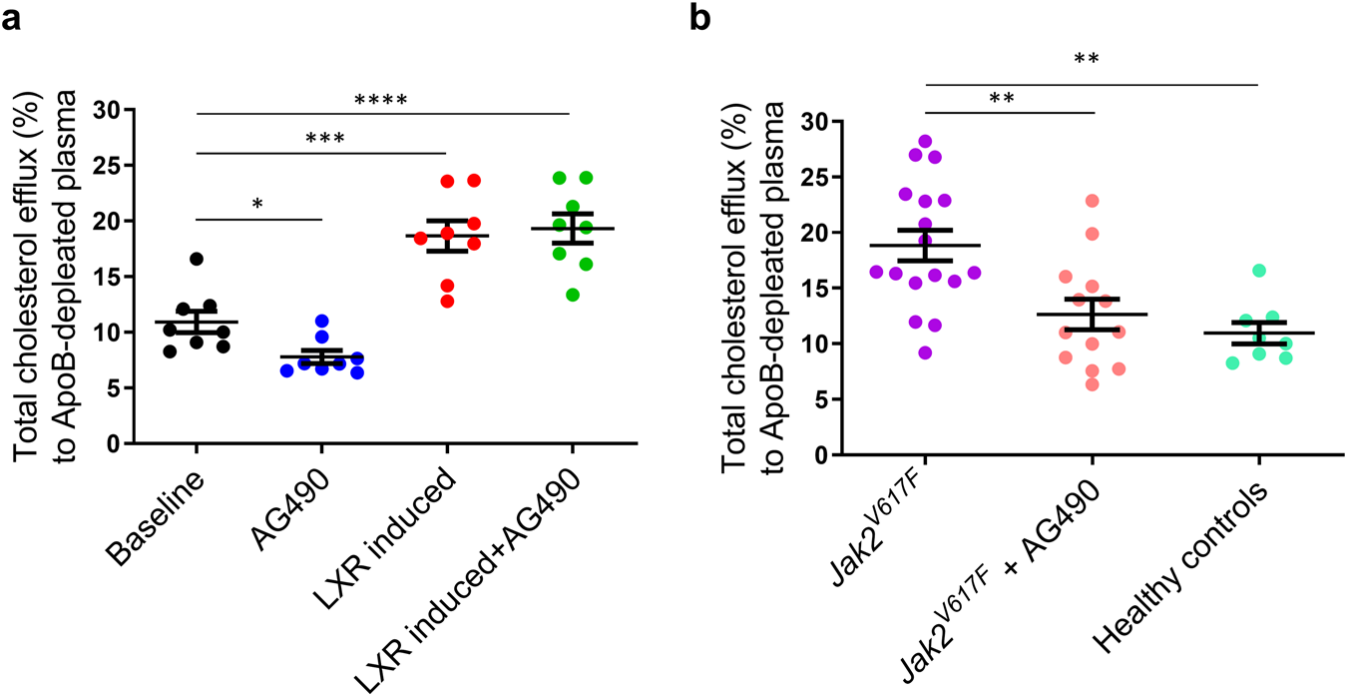
Cholesterol efflux from macrophages of individuals harboring the *Jak2*^*V617F*^ mutation. **a** Cholesterol efflux from blood monocyte-derived macrophages of healthy donors measured at baseline and after treatment with AG490, TO901317, or both (*n* = 8/group), **b** and from individuals harboring the *Jak2*^*V617F*^ mutation at baseline and after treatment with AG490 (*n* = 8–17/group). Statistical analysis: one-way ANOVA with Tukey’s multiple comparisons were performed. Data are presented as the mean ± SEM, *P < 0.05, **P < 0.01, ***P < 0.001, ****P < 0.0001.

Some individuals with myeloproliferative disorders harbor the activating *Jak2*^*V617F*^ mutation. This has recently been linked to atherosclerotic cardiovascular disease in humans^24^ and mice that were previously associated with increased inflammation and defective efferocytosis^11^. We, therefore, wanted to assess whether cholesterol efflux was affected in macrophages of these individuals. To this end, we first assessed cholesterol efflux from macrophages differentiated from blood monocytes of healthy donors and their response to JAK2 inhibition. Similar to our findings in M-Jak2 KO mice, cholesterol efflux was decreased when macrophages were treated with a JAK2 inhibitor, AG490. On the other hand, cholesterol efflux was augmented with an LXR agonist, TO901317; and AG490-induced defect was overcome when co-treated with TO901317 (Fig. 6a). We next assessed for the effects of the activating Jak2 mutation on cholesterol efflux by examining macrophages derived from peripheral monocytes of individuals with an acquired *Jak2*^*V617F*^ mutation. Remarkably, these mutation-harboring macrophages showed enhanced efflux compared to macrophages from healthy individuals (Fig. 6b). Moreover, inhibiting JAK2 with AG490 in macrophages harboring the *Jak2*^*V617F*^ mutation abolished their enhanced cholesterol efflux to levels similar to healthy individuals (Fig. 6b). Taken together, these data show that inhibiting JAK2 attenuates cholesterol efflux, and that an activating Jak2 mutation increases cholesterol efflux, supporting a critical role of Jak2 in modulating cholesterol efflux both in mice and humans.

## Discussion

Atherosclerosis and its consequences, cardio- and cerebrovascular diseases, continue to be the leading causes of death worldwide despite recent advances in treatment^25^. In this study, we examined the specific role of macrophage Jak2 in atherosclerosis. JAK2 is a pivotal molecule in inflammatory and metabolic signal transduction pathways. Using mice with M-Jak2 deletion, and mice treated with ruxolitinib, a clinically available JAK2 inhibitor, we showed that M-Jak2 is essential for attenuation of atherosclerosis. As such, mice deficient in M-Jak2, as well as mice that were treated with JAK2 inhibitor, developed accelerated atherosclerosis. We also found that conventional cardiovascular risk factors, such as obesity, insulin resistance, glucose intolerance, or dyslipidemia, did not likely play a role in the pathogenesis of our findings. Rather, the increased plaque burden seen in our M-Jak2 mice resulted from a defect in cholesterol efflux. This defect was overcome by LXR-mediated agonism that is known to transcriptionally induce both *ABCA1* and *ABCG1*.

We have previously shown that M-Jak2 deficiency in mice fed a high-fat diet protects them from obesity and systemic inflammation^10^. These findings, together with the putative role of Jak2 in mediating inflammation, and along with the current understanding that atherosclerosis is a chronic inflammatory condition^1^, led us to hypothesize that M-Jak2 deficiency would provide protection against atherosclerosis progression. Others have also recently shown reduced atherosclerosis in mice and rabbits treated with a selective JAK2 inhibitor (TG101348) or ruxolitinib^26^. Surprisingly, we found that M-Jak2 deficiency led to accelerated atherosclerosis after a high cholesterol-containing diet. Since conventional risk factors, such as obesity, dyslipidemia, and diabetes, could not account for this phenotype, we further delved into examining various macrophage functions that are relevant for atherosclerosis. Indeed there are a number of clinical trials where favorable metabolic parameters such as glucose homeostasis did not extend to cardiovascular benefit^27^.

Atherosclerosis development and progression is a dynamic complex process involving numerous cell types^26^. Specifically, in the plaque microenvironment, macrophages play a major role in both plaque initiation and progression. Monocytes are recruited into the plaque, where they become macrophages and proliferate and secrete mediators that can affect other cells, take up and efflux cholesterol, undergo cell death within the plaque, and clear dead cells and debris by efferocytosis^26^. All these processes are orchestrated by complex signal transduction pathways within each of the cell types contributing to the atherosclerotic process. We examined monocyte recruitment and plaque macrophage proliferation in vivo but found no difference between M-Jak2 WT and KO mice. We also found no difference in cholesterol uptake by macrophages. Moreover, pro- and anti-inflammatory gene expression and response to oxLDL and LPS was not predominantly different in BMDM from M-Jak2 KO and WT mice. Several signal transduction pathways contribute to macrophage biology within the plaque. For example, p38 MAPK is involved in the prevention of apoptosis and necrosis^28^, AKT can regulate macrophage polarization^29^, and wnt/β-catenin modulates the inflammatory response^30^. Yet, the in vivo role of the JAK-STAT pathway in macrophages in the context of atherosclerosis was unclear.

In vitro studies have shown JAK2 in macrophage to be activated by ABCA1, with a dual function of enhancing cholesterol efflux and modulating inflammation^8,15,16^. Whereas previous in vitro studies in cell lines found that the interaction between ApoA1 and ABCA1 activates JAK2, and this activation results in an increase in anti-inflammatory M2-like cytokines and a decrease in pro-inflammatory M1-like cytokines^8^, our ex vivo findings in BMDM from M-Jak2 KO mice showed mixed results with some increase in M1 cytokines compared to WT controls. With regards to M2 cytokines, the results were also mixed with some increased, some unchanged and some decreased in *Jak2*-deficient macroaphges. These results are in keeping with the literature, with evidence suggesting JAK2 mediates an anti-inflammatory effect^8,31,32^, while other sources show the opposite, whereby JAK2 inhibition attenuates inflammation in certain conditions^33^.

ABCA1 is a cell membrane protein that is abundant in macrophages. It is best known for its role in cholesterol efflux to ApoA1 and lipid-poor HDL as the first step of reverse cholesterol transport that is critical in atherosclerosis^34,35^. Indeed, increasing cholesterol efflux by inhibition of ABCA1 protein degradation was shown to reduce atherosclerosis in mice^36^, and efflux capacity via ABCA1 was shown to inversely correlate with cardiovascular events in humans^6,37^. In experimental cell lines, ApoA1 binding to ABCA1 can stimulate JAK2 autophosphorylation, that in turn enhances the interaction between ApoA1 and ABCA1^15^, thus increasing its capacity to efflux cholesterol. Treating macrophages with a JAK2 inhibitor did not alter membrane ABCA1 content, yet, in line with our results, it decreased cholesterol efflux^15^, emphasizing the important role of JAK2 in modulating lipid transport properties of ABCA1^16^.

We assessed cholesterol efflux from BMDM in several different conditions. After labeling the cells with ^3^H-cholesterol, we measured efflux to either ApoB-depleted plasma, which contains all sizes of HDL or ApoA1, to capture efflux that occurs via ABCA1, as well as ABCG1 and SRB1. We also measured efflux to purified ApoA1, which occurs as the first step of efflux via ABCA1. In all conditions, we found a consistently lower efflux from BMDM of M-Jak2 KO mice, highlighting the importance of *Jak2* in this process. To further show that the decrease in efflux is indeed a consequence of *Jak2* deficiency, we took a pharmacologic approach and treated WT BMDM with AG490, a JAK2 inhibitor. Again, we found a significant reduction in efflux. Importantly, no further reduction in efflux was observed from BMDM of M-Jak2 KO mice, showing the specificity of the inhibitor to JAK2 in regulating efflux. We further stimulated the cells with cAMP, which increases the transcription and phosphorylation mainly of ABCA1^34,38^. The defect in efflux persisted with Jak2 deficiency, highlighting that inducing ABCA1 was insufficient to overcome the defect in efflux.

To understand the mechanistic role of myeloid *Jak2* in atherosclerosis, we treated BMDM with an LXR agonist, TO91317 that increases the transcription of ABCA1 and ABCG1^39–41^. This treatment abolished the defect in efflux in M-Jak2 KO BMDM, overcoming the consequences of *Jak2* deficiency, highlighting its essential role in promoting cholesterol efflux. Finally, we subjected M-Jak2 KO and WT mice on HCD with TO901317 or vehicle for 3 weeks and found that plaque burden was attenuated in M-Jak2 KO mice exposed to TO901317, showing the critical role of cholesterol efflux that was regulated by JAK2 in determining atherosclerosis progression^40,41^.

In order to extend our findings in mice to humans, we examined the effects of JAK2 inhibition as well as LXR agonism in macrophage derived from healthy individuals. Similar to mouse macrophages, efflux from human macrophages decreased when JAK2 was inhibited, and increased when LXR was induced. Furthermore, concomitant treatment of LXR agonist with JAK2 inhibitor abolished the defect in efflux mediated by JAK2 inhibitor, recapitulating our results from experiments using mouse BMDM, showing normalization of efflux in M-Jak2 KO with LXR agonist.

Jak2 deletion in mouse hematopoietic stem cells leads to embryonic lethality, due to the development of severe anemia and thrombocytopenia^42^. Contrastingly, *Jak2*^*V617F*^ mutation has been implicated in clonal hematopoiesis and increased risk for adverse cardiovascular outcomes. In addition to hyperviscosity that may cause thrombosis, mice harboring the *Jak2*^*V617F*^ mutation in hematopoietic cells are prone to develop atherosclerosis due to increased hematopoiesis and neutrophil infiltration in early lesions, along with defective efferocytosis and increased inflammation in advanced lesions^11^. Of note, the authors in this study did not examine cholesterol efflux from macrophages. More recently, *Jak2*^*V617F*^ mutation in macrophages has been shown to promote atherosclerosis through activating AIM2 inflammasome^43^. The *Jak2*^*V617F*^ mutation is an activating mutation that renders the kinase constitutively active^44^. As such, one can speculate that efflux via ABCA1 is increased, with increase ABCA1 phosphorylation. Indeed, comparing cholesterol efflux from macrophages of individuals carrying this activating mutation to healthy volunteers, we found that these subjects have an increased efflux, which might serve as an athero-protective mechanism. Treating cells with JAK2 inhibitor resulted in a reduction in efflux to a level similar to that of healthy subjects, thereby abolishing the potential cardiovascular protection that may be provided via their JAK2 activation and increasing their cardiovascular risk further.

There have been a few recent reports that found reduced plaque burden using JAK2 inhibitors in different animal models. Tang et al. found reduced plaque burden in the descending aorta in mice treated with a selective JAK2 inhibitor, TG101348^45^. In this study, a different JAK2 inhibitor was used and was given by oral gavage, which may result in different pharmacodynamics compared to our study where we incorporated the drug in diet. Importantly, only descending aortas were examined in their study, where we also did not find a difference in our mice following exposure to ruxolitinib. The aortic arch, where we noted a striking difference was not examined by this group. Yang et al. showed that ruxolitinib reduced atherosclerotic plaques in rabbits treated with high-fat diet^26^. In this study, a mechanical insult was used to induce atherosclerosis which is very different from the genetic model of chronic atherosclerosis development that we used in this study and recapitulates many features of human atherosclerosis. It is also well known that different animal models have different lipid and lipoprotein biology which may account for the differences in atherosclerosis susceptibility in response to JAK2 inhibitor. This group also did not examine the aortic arch or root where we found the most striking differences. Overall, we show in this study using multiple approaches, including specific genetic deletion of Jak2 in myeloid cells that leads to accelerated atherosclerosis that is recapitulated by systemic JAK2 inhibition in vivo. Our in vivo results are corroborated by our findings in vitro in BMDM as well as in blood monocyte-derived macrophages of individuals in response to JAK2 inhibition or in subjects carrying the *Jak2*-activating mutation that together support the predominant role of JAK2 in enhancing cholesterol efflux from macrophages that likely contribute to accelerated atherosclerosis.

In conclusion, we showed in this study that M-Jak2 deficiency causes accelerated atherosclerosis largely due to defects in cholesterol efflux. These results have clinical implications in individuals with myeloproliferative and autoinflammatory disorders that require long-term treatment with JAK2 inhibitors. These diseases are already associated with increased cardiovascular risk. As such, cautious clinical assessment is needed for their cardiovascular risk management that may be negatively impacted by JAK2 inhibition.

## Methods

### Generation of ApoE-null myeloid Jak2-KO mice

Myeloid-specific Jak2 KO mice were generated by breeding mice with the *Jak2* gene flanked by *loxP* sites (*Jak2*^*fl/fl*^)^46^ with mice expressing Cre recombinase under the control of the LysM promoter. Jak2 floxed mice were generously provided by Kay-Uwe Wagner (Wayne State University, Detroit, MI, USA) and LysM-cre transgenic mice were purchased from Jackson Laboratory (Bar Harbor, ME, USA). The resulting LysM-cre^+^*Jak2*^*+/fl*^ mice were intercrossed to generate LysM-cre^+^*Jak2*^*fl/fl*^ mice. To generate ApoE-null myeloid Jak2-deficient mice, LysM-cre^+^*Jak2*^*fl/fl*^ mice were crossed with *Apoe*^*–/–*^ mice (The Jackson Laboratory, B6.129P2-Apoetm1Unc/J) resulting in LysM-Cre^+^*Jak2*^*+/fl*^*Apoe*^*+/−*^ mice. These mice were intercrossed to generate LysM-Cre^+^*Jak2*^*fl/fl*^*Apoe*^*−/−*^ (referred herein as M-Jak2 KO) and LysM-Cre^+^*Jak2*^*+/+*^*Apoe*^*−/−*^ littermate controls (referred herein as M-Jak2 WT).

Mice were housed in a temperature-controlled pathogen-free animal facility with a 12-h light and dark cycle with free access to water and food. Male and female mice were used for experiments and were fed a standard rodent chow diet (5% fat; Harlan Teklad) until 6 weeks of age followed by an atherogenic diet containing 0.2% cholesterol (TD88137, Harlan Laboratories) for 12 weeks. Data from males and females were analyzed separately. Another male cohort were fed atherogenic diet for 3 weeks starting at 6 weeks of age. A third cohort of male ApoE-null mice that were fed with either atherogenic diet (ENVIGO, TD88137) or same atherogenic diet containing 2 g/kg of ruxolitinib (ENVIGO, TD180522, Western Ruxolitinib Diet), similar to previously described protocol^47^. All animal experimental protocols were approved and performed in accordance with the guidelines of the Canadian Council on Animal Care and regulations established by the Toronto General Hospital Research Institute Animal Care Committee (AUP 2862).

### In vivo metabolic analyses

Random blood glucose measurements were measured as previously described^48^. Intraperitoneal glucose (i.p., 1 g/kg) and insulin (0.75 U/kg) tolerance tests were performed as previously described^48^.

### Atherosclerotic plaque assessment and quantification

Atherosclerotic plaque burden in the descending aorta was assessed distal to the left subclavian artery to the iliac bifurcation as previously described^49^. Mice were perfused with PBS followed by 4% paraformaldehyde (PFA) before extraction of the aorta including the aortic arch to the iliac bifurcation and the descending aorta was severed from the aortic arch for a separate analysis. Adipose tissues were removed from the aorta before staining with Oil-red-O (ORO) (Sigma-Aldrich). Stock solution was 0.3 g/10 mL isopropanol with working solution comprising of a 3:2 ratio of stock ORO to water. Aortas were stained for 30 min followed by 2 washes with 60% isopropanol. The aortic arch and descending thoracic aorta were pinned for en face plaque area measurement and images were captured using a stereomicroscope (Leica S9). Sagittal aortic arch and root sections were stained with hematoxylin and eosin (H&E) and lesion area was measured as previously described^49^. The extent of lesion development was defined as the percentage of ORO-positive plaque area per total aortic surface area and was quantified using ImageJ (National Institutes of Health, Bethesda, MD, USA). En face arches were stained with ORO and plaques quantified as area in µm^2^.

### Histological assessment

Aortic arches were isolated, fixed in 4% PFA in 0.1 M phosphate buffer saline (PBS; pH 7.4) for 2 days, and then processed to paraffin blocks (UHN Pathology Research Program, Toronto, ON). Tissue sections were stained with hematoxylin and eosin (H&E) as described^49^. The combined Masson trichrome and Verhoeff Van Gieson stain of the lesser curvature of the aortic arch were done in the STARR facility as described^50^. Quantification was performed using ImageJ software.

### En face BrdU and immunofluorescent staining

For BrdU labeling experiments, mice received a single 0.2 mL i.v. injection of 2 mg BrdU in PBS, as described^51^. Aortas were harvested after 3 or 24 h. En face immunostaining was performed as previously described^51^. Mice were perfused with PBS, followed by 4% paraformaldehyde (PFA). The aortic arch was harvested, the periadventitial fat was removed and the aorta was fixed for 1 h in 4% PFA. After permeabilization with 0.5% Triton X-100 and 10% DMSO for 5 min at room temperature (RT), the aortic arch was incubated with Alexa Fluor^®^ 647-conjugated anti-CD68 antibody (BioLegend) or Alexa Fluor^®^ 647-conjugated anti-CD45 (BioLegend) and biotin-conjugated anti-BrdU antibodies overnight at 4 °C, followed by Cy^TM^3-Streptavidin. Nuclei were stained with Hoechst 33342. The aorta was opened and mounted on a glass slide. En face immunofluorescence confocal images were obtained using Nikon A1R confocal microscope (Nikon, Tokyo, Japan) equipped with ×40 oil objectives. Every 2 mm z-stack slices were aligned and compressed to create a maximum intensity projection image. ImageJ software was used for analysis.

### Flow cytometry

For circulating leukocyte analysis, blood was collected from mice into heparin-coated capillary tubes and RBCs were lysed with RBC lysis buffer (BioLegend). Cells were resuspended in FACS buffer and allowed to block non-specific binding in Fc receptor blocking solution. Cells were incubated on ice for 30 min with fluorophore-conjugated primary antibodies: CD45 (EF-450), CD11b (M1/70), CD3e (145-2C11), B220 (30-F11), Ly6G (1A8), Ly6C (AL-21), and CD115 (AFS98) using recommended dilutions from the supplier (BD Pharmingen). Data were acquired on a Fortessa or LSRII flow cytometer (BD Biosciences, San Jose, CA, USA) and analyzed with FlowJo software (Tree Star, Ashland, OR, USA).

### BMDM isolation and polarization experiments

BM was collected from the long bones of M-Jak2-WT and KO mice, at the age of 6–8 weeks and cultured in the presence of 40 ng/mL M-CSF in DMEM supplemented with 10% FBS and antibiotic for the first 3 days. Subsequently, cells were cultured with 20 ng/mL M-CSF for 5–7 days. The medium and cytokines were replaced every 2–3 days.

BMDM were loaded with oxLDL (Kalen Biomedical, 100 μg/mL-24 h), and subsequently stimulated with LPS (10 ng/mL, Ultrapure LPS, InvivoGen) for 6 h as described^13^. RNA isolation and RT-qPCR were performed as described^13^.

For polarization, BMDM were stimulated with LPS (10 ng/mL) or IL4 (10 ng/ml, Pepro Tech) for 24 h as described^52^. RNA was isolated for RT-qPCR analysis.

### Quantitative RT-PCR

Total RNA from BMDM was isolated using Trizol (Ambion, Carlsbad, CA, USA) as per the manufacturer’s protocol with the following changes. After adding isopropanol, the suspension was stored at −20 °C overnight. Next day, the pellet was washed with 75% ethanol. 1 µg RNA was treated with 1U DNase I (Invitrogen) with 10× DNase I Reaction Buffer (1× final concentration; Invitrogen), incubated for 15 min at room temperature (RT) and then inactivated at 75 °C for 5 min. Subsequently, 0.5 nM dNTP and 200 ng of random primers (Invitrogen) were added to the reaction mixture, incubated at 65 °C for 5 min followed by a quick chill on ice. Then 5× First-Strand Buffer (1× final concentration; Invitrogen), 0.01 M DTT (Invitrogen) and 40U RNase OUT (Invitrogen) were added to the reaction mixture and incubated at 37 °C for 2 min. RNA was then reverse-transcribed with 200 U of M-MLV enzyme (Invitrogen) using the following protocol: 25 °C for 10 min, 37 °C for 50 min, 70 °C for 15 min and held at 4 °C. qPCR was performed under standard conditions using SYBR Green master mix on a 7900HT Fast-Real-Time PCR System (Applied Biosystems, Carlsbad, CA, USA). Each sample was run in triplicates in 10 µL volume. The relative mRNA abundance of each gene was normalized to the expression level of the housekeeping gene 18S and calculated by a standard curve. Primer sequences are listed in Supplementary Table S1.

### Western blotting

BMDM and tissue lysates were mechanically homogenized in ice-cold RIPA buffer (Sigma-Aldrich) with a protease and phosphatase inhibitor (CST#5872 S) and centrifuged for 10 min at 14,000 × *g* and 4 °C. In total, 60 μg of total protein from tissue or cell lysate was run on an 8% sodium dodecyl sulfate-polyacrylamide gel electrophoresis (SDS-PAGE). After electrophoresis, proteins were transferred to an ethanol-hydrated PVDF (polyvinylidene fluoride) membrane for 10 min using the Trans-Blot Turbo Transfer System (Bio-Rad) and blocked with 5% non-fat dry milk or bovine serum albumin (BSA; for phosphorylated proteins) for 1 h at RT. Membranes were probed overnight at 4 °C with a primary antibody: total JAK2 (CST#3230; 1:1000), total ABCA1 (NB400-105; 1:500), total SRB1 (NB400-104; 1:1000), total ABCG1 (NB400-132; 1:500), and β-actin (CST#4967; 1:1000). After overnight incubation, membranes were washed three times with TBST for 10 min and then incubated for 1 h at RT with an anti-rabbit IgG horseradish-peroxidase (HRP)-conjugated secondary antibody (CST#7074; 1:5000). Membranes were then incubated in chemiluminescent ECL-plus reagent (PerkinElmer Inc., Waltham, MA, USA) at RT and then imaged using MicroChemi 4.2 (DNR Bio-Imaging Systems, Mahale HaHamisha, Jerusalem, Israel). Protein loading was confirmed by probing for β-actin. The quantification of the western blots was performed by densitometry using ImageJ.

### [^3^H]-cholesterol uptake assay

BMDM were incubated with 2μCi/mL[^3^H]-cholesterol (PerkinElmer) in DMEM containing 1% FBS. Media were collected at times 0, 60, 120, 180, 240, 300, and 360 min and were placed at 4 °C. Cells were washed and incubated with 0.1 N NaOH overnight and collected the next day; 100 μL of lysed cells were added into 5 mL of scintillation fluid (Ecolite + , MP Biomedicals, Santa Ana, CA). Finally, media were spun at 4 °C for 10 min at 1500 × *g* and 200 μL were added to scintillation fluid. The radioactivity was counted using a Tri-Carb 2800TR Liquid Scintillation Analyzer (PerkinElmer). Cells were washed with media prior to each step. % cholesterol efflux uptake was calculated as cells[^3^H]cpm/(medium[^3^H]cpm + cells[^3^H]cpm).

### Cholesterol assay by BODIPY

BMDM were incubated with either 50 µg/mL of LDL (Kalen biomedical) or OxLDL (Kalen biomedical) for 24 h, and then washed. Subsequently, BMDM were incubated with 0.01 mg/mL labeled BODIPY™ 493/503 (Molecular Probes) for 30 min at 37 °C, followed by extensive washes (five times) with warm PBS (37 °C). Cells were incubated in serum-free DMEM and imaged live immediately after BODIPY labeling using a confocal microscope. Confocal images were acquired using a Yokogawa CSU10 spinning disk system (Quorum Technologies Inc., Guelph, ON). Images were acquired using a ×63/1.4 NA oil objective or ×25/0.8 NA water objective (ZEISS, Germany), as indicated, with an additional ×1.5 magnifying lens. For live experiments, cells were maintained at 37 °C using an environmental chamber (Live Cell Instruments, Korea). Routine analyses were done using Volocity software (PerkinElmer, Woodbridge, ON).

### Ex vivo HDL cholesterol efflux studies

Cholesterol efflux experiments were performed as previously described with the following modifications^53^. For the total cholesterol efflux assay, BMDM was labeled for 24 h with 2μCi/mL [^3^H]-cholesterol (PerkinElmer) and 2 μg/mL acyl-coenzymeA cholesterol acyltransferase (ACAT) inhibitor (Sandoz, Sigma) in DMEM containing 1% FBS. The following day, either 20 µg/mL of ApoA1 (Academy Bio-Medical Company, Houston, TX) or 2.8% apoB-depleted plasma (courtesy of Dr. Jacques Genest)^53^ and 2 μg/mL ACAT inhibitor in serum-free DMEM was added and incubated for 5 h. Subsequently, media were collected and placed at 4 °C. Cells were washed and incubated with 0.1 N NaOH overnight and collected the next day; 100 μL of cells were added into 5 mL of scintillation fluid (Ecolite + , MP Biomedicals, Santa Ana, CA). Finally, media were spun at 4 °C for 10 min at 1500 × *g* and 200 μL of media were added to scintillation fluid. The radioactivity was counted using a Tri-Carb 2800TR Liquid Scintillation Analyzer (PerkinElmer). Cells were washed with media prior to each step. For the cAMP-induced cholesterol efflux assay, cells were labeled for 24 h. The following day, cells were stimulated with 0.5 mM 8-Br-cAMP (Sigma, Oakville, Canada) in DMEM with 0.2% bovine serum albumin (BSA, Sigma, Oakville, Canada) for 18 h followed by treatment with either ApoA1 or apoB-depleted plasma^53^ and ACAT inhibitor in serum-free DMEM, and incubated for 5 h. The media and cells were collected as described above, and radioactivity was counted using the scintillation analyzer. For the efflux experiments with AG490 (Sigma-Aldrich), cells were treated with 60 µM AG490 for 24 h prior to efflux as well as during efflux. For the efflux experiment with TO901317 (Selleck Chemicals, Houston, TX), cells were treated with 5 µM TO901317 for 24 h prior to efflux. % cholesterol efflux was calculated as medium[^3^H]cpm/(medium[^3^H]cpm + cells[^3^H]cpm). All experiments were performed in triplicates.

### Colony-formation assays

BM cells from M-Jak2 KO and WT mice were obtained by flushing the femur with Iscove’s Modified Dulbecco’s Medium (IMDM, Wisent) and filtering the cell suspension through a 40-µm mesh. Cells were then counted with trypan blue. In all, 2 × 10^4^ cells/mL/mouse were cultured in duplicates in Methocult GF (Stem Cell Technologies). Cells were incubated at 37 °C, 5% CO_2_ with 95% humidity for 5–7 days. After the incubation, the number of colonies containing 50 or more cells was counted on an inverted microscope.

### Peripheral blood monocyte isolation

Heparinized peripheral blood from healthy donors was diluted in 1:1 ratio with PBS. 30 mL of the mixture was added to 15 mL of Lympholyte (Lympholyte-H Cell Separation Media, Cedarlane, Burlington, Canada) and spun at 800 × *g* for 20 min. The buffy coat (monocyte/macrophage layer) was then collected using an eyedropper pipette and mixed with 1×HBSS, and spun at 500 × *g* for 10 min. HBSS was then aspirated and cells were resuspended in RPMI and seeded on 24-well plates. One hour after seeding, media was replaced with RPMI with 10 ng/ml M-CSF (Pepro Tech, NJ, USA). The medium was replaced after 2–3 days. All protocol was approved by the Hospital for Sick Children Ethics Approval 1000060065 and written consent was obtained for all participants. Frozen mononuclear cells from peripheral blood of consented patients with myeloproliferative neoplasms (PMN) harboring the *Jak2*^*V617F*^ mutation were obtained from the biobank of Princess Margaret Cancer Centre, Toronto. Patient information is listed in Supplementary Table S2. The research protocol was reviewed and approved by the University Health Network Research Ethics Board (01-0573).

### Statistics and reproducibility

All data are presented as mean ± standard error of mean (SEM). All statistical parameters are described in figure legends. Comparisons between two groups were analyzed by two-tailed unpaired *t* test. For comparison among multiple groups, one-way or two-way ANOVA with Tukey’s post hoc multiple comparison test or multiple *t* tests with Holm-Sidek correction were performed. All statistical analysis was performed using GraphPad Prism (La Jolla, CA). A value of *P* < 0.05 was considered statistically significant. Samples sizes were predetermined based on statistical power calculations. For experiments with a high variability, *n* < 10; for assays with a low variability, *n* < 10.

### Reporting summary

Further information on research design is available in the Nature Research Reporting Summary linked to this article.

## Supplementary information

Supplementary Information

Description of Additional Supplementary Files

Supplementary Data 1

Reporting Summary

## Data Availability

The authors declare that all data supporting the findings of this study are available within the paper and its supplementary information files. Uncropped western blot images are provided in Supplementary Fig. S14. The source data behind the graphs in the paper can be found in Supplementary Data 1.
