# Supplementary material for: Macrophage Jak2 deficiency accelerates atherosclerosis through defects in cholesterol efflux
Source: Commun Biol. 2022 Feb 15;5:132. doi: 10.1038/s42003-022-03078-5 (PMC8847578; doi:10.1038/s42003-022-03078-5)
Supplement: Supplementary file 1 — Supplementary Information [file 42003_2022_3078_MOESM1_ESM.pdf]

Macrophage Jak2 deficiency accelerates atherosclerosis through defects in cholesterol efflux

Idit Dotan, Jiaqi Yang, Jiro Ikeda, Ziv Roth, Evan Pollock-Tahiri, Harsh Desai, Tharini Sivasubramaniyam, Sonia Rehal, Josh Rapps, Yu Zhe Li, Helen Le, Gedaliah Farber, Edouard Alchami, Changting Xiao, Saraf Karim, Marcela Gronda, Michael F. Saikali, Amit Tirosh, Kay-Uwe Wagner, Jacques Genest, Aaron D. Schimmer, Vikas Gupta, Mark D. Minden, Carolyn L. Cummins, Gary F. Lewis, Clinton Robbins, Jenny Jongstra-Bilen, Myron Cybulsky and Minna Woo

Supplementary Figures

Figure S1

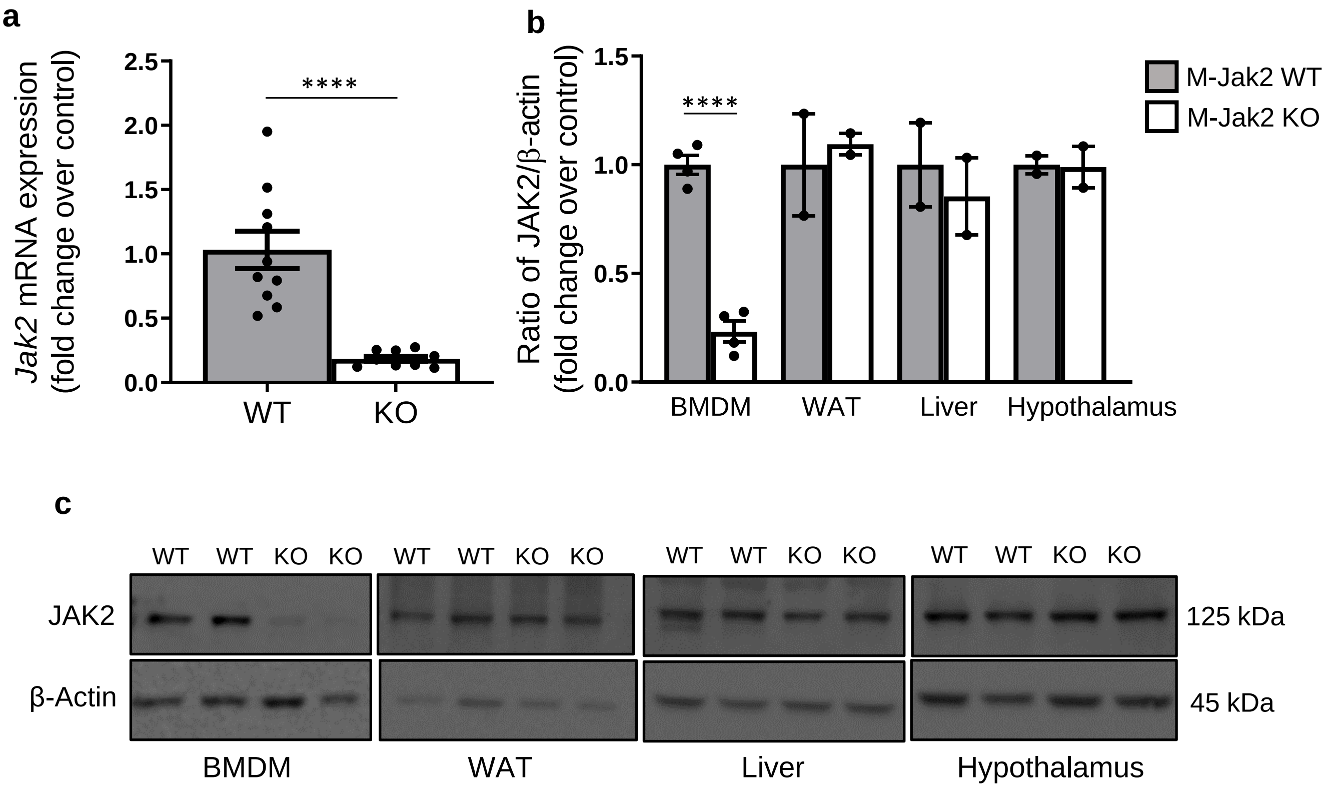

Figure S1. Jak2 gene and protein expression in M-Jak2 KO and WT mice. (a) Quantification of Jak2 gene expression in BMDM from M-Jak2 KO and WT mice (n=9-10/genotype). **(b)** Quantification of total JAK2 protein content in BMDM (n=4/genotype), white adipose tissue (WAT) (n=2/genotype), liver (n=2/genotype) or hypothalamus (n=2/genotype) from M-Jak2 KO and WT mice, normalized to β-actin. **(c)** Western blot images of total Jak2 content in BMDM, WAT, liver and hypothalamus. Statistical analysis: Two-tailed unpaired t-tests were performed. Data are presented as the mean ± SEM, ****$p<0.0001$.

Figure S2

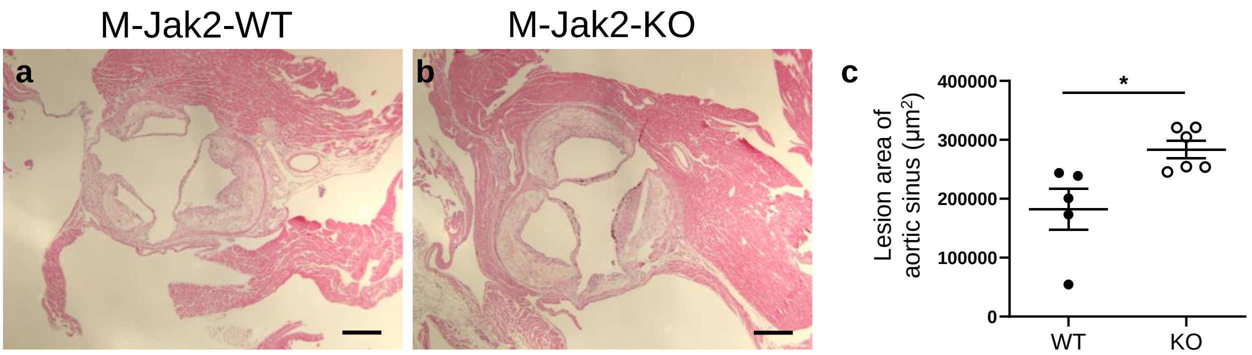

Figure S2. Accelerated atherosclerosis in the aortic sinus of M-Jak2 KO mice. (a and b) Representative images of H&E staining of aortic sinus sections from M-Jak2 KO and WT controls fed 16 weeks of HCD. Scale bar, 200 µm. **(c)** Quantification of atherosclerotic plaque area in the aortic sinus from M-Jak2 KO and WT controls fed 16 weeks of HCD (n=5-6/genotype). Statistical analysis: Two-tailed unpaired t-tests were performed. Data are presented as the mean ± SEM. *$p < 0.05$.

Figure S3

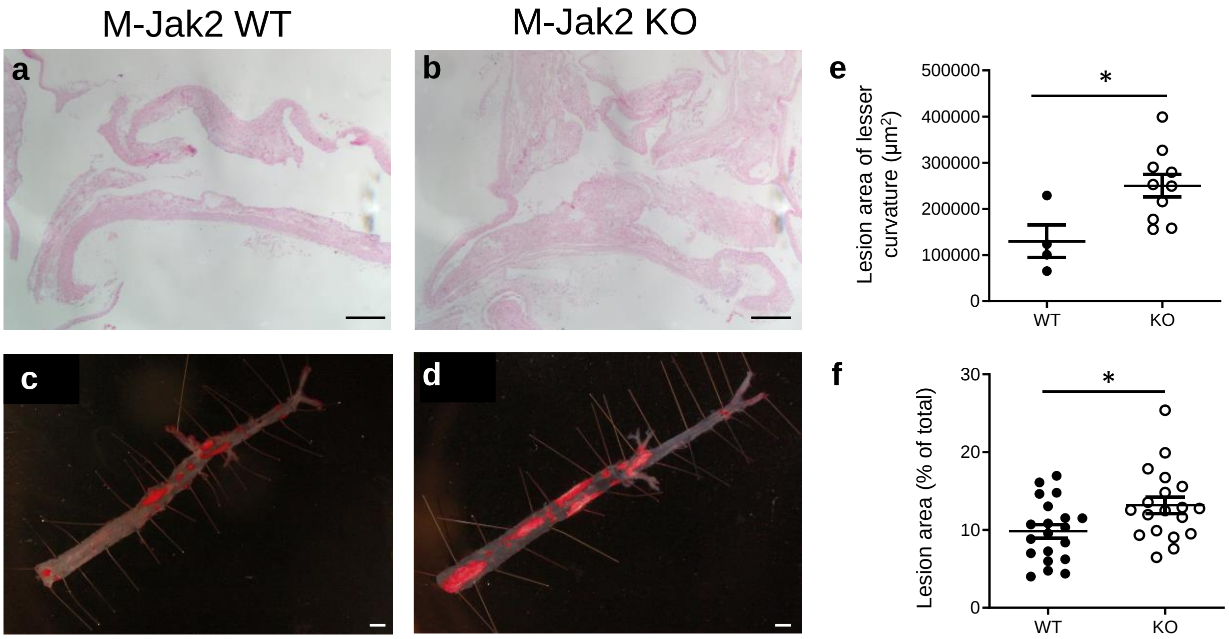

Figure S3. Atherosclerosis in M-Jak2-deficient female mice. (a-d) Representative images of H&E staining of aortic arch sections (a and b) and *en face* Oil-red-O staining of atherosclerotic plaque area in the descending aorta (c and d) from M-Jak2 KO and WT control female mice fed 16 weeks of HCD starting at 6 weeks of age. Scale bar 200 µm. **(e and f)** Quantification of atherosclerotic plaque area in aortic arch (e) and descending aorta (f) from M-Jak2 KO and WT control female mice fed 16 weeks of HCD starting at 6 weeks of age (n=4-20/genotype). Statistical analysis: Two-tailed unpaired t-tests were performed. Data are presented as the mean ± SEM. *$p < 0.05$

Figure S4

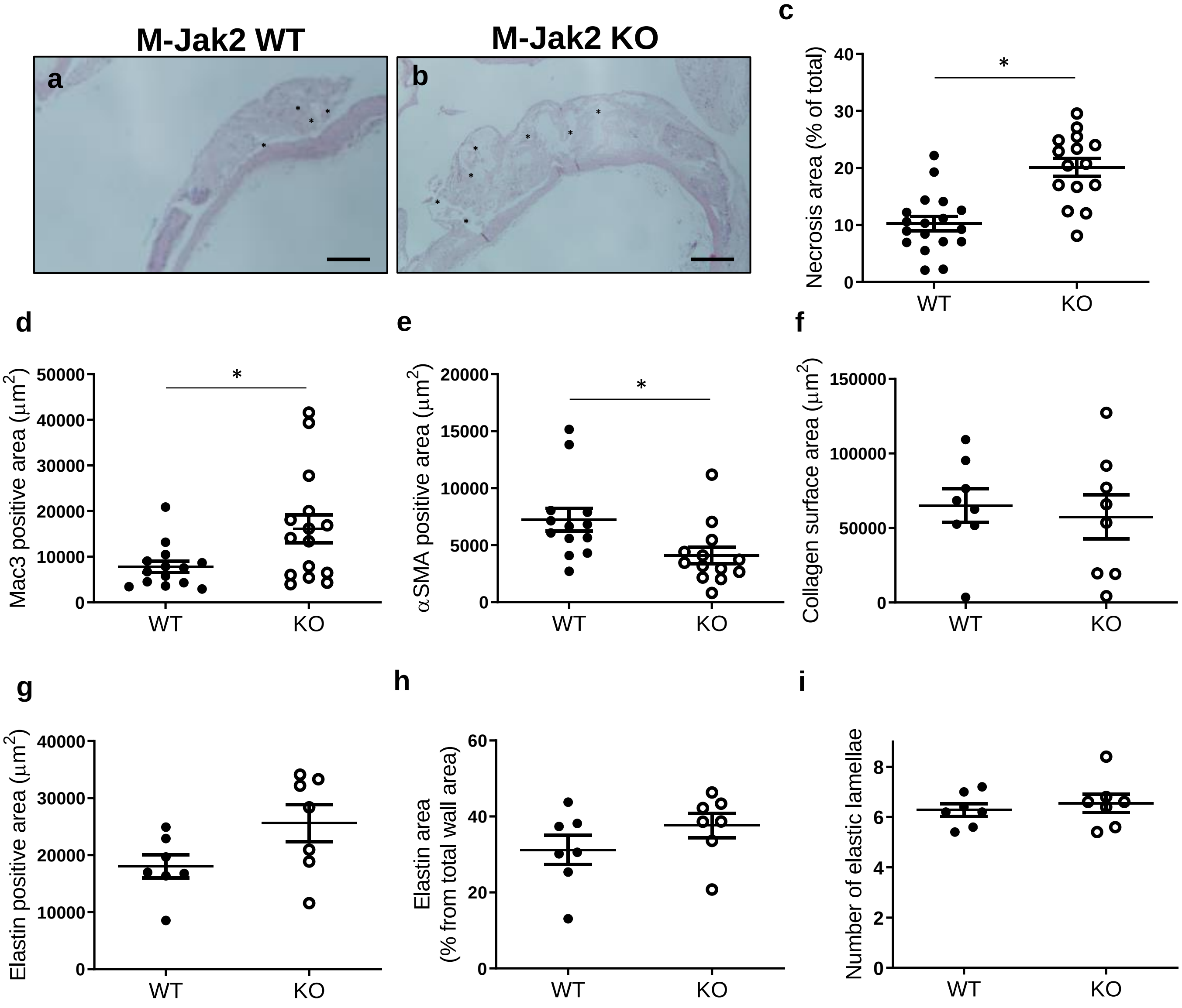

Figure S4. Plaque characterization in the lesser curvature of the aortic arch. **(a and b)** Representative images of the plaque from sagittal sections of the lesser curvature of aortic arch of M-Jak2 KO and WT mice fed 16 weeks of HCD staining with H&E showing necrotic core areas (*). Scale bars, 100 µm. **(c)** Quantification of necrotic core areas expressed as a percentage of total plaque area (n=15-18/genotype). **(d)** Quantification of Mac3 positive surface area within the plaque (µm^2) (n=14-15/genotype). **(e)** Quantification of α-smooth muscle actin positive surface area within the plaque (µm^2) of the consecutive slide (n=12-13/genotype). **(f)** Quantification of collagen surface area within the plaque (µm^2) of the consecutive slide using Masson trichrome stain (n=8/genotype). **(g-i)** Quantification of elastin surface area (µm^2), percentage of total wall area, and number of elastic lamella within the plaque of the consecutive slide using Vehoeff-Van Giesen stain (n=7/genotype). Statistical analysis: Two-tailed unpaired t-tests were performed. Data are presented as the mean ± SEM, *$p<0.05$.

Figure S5

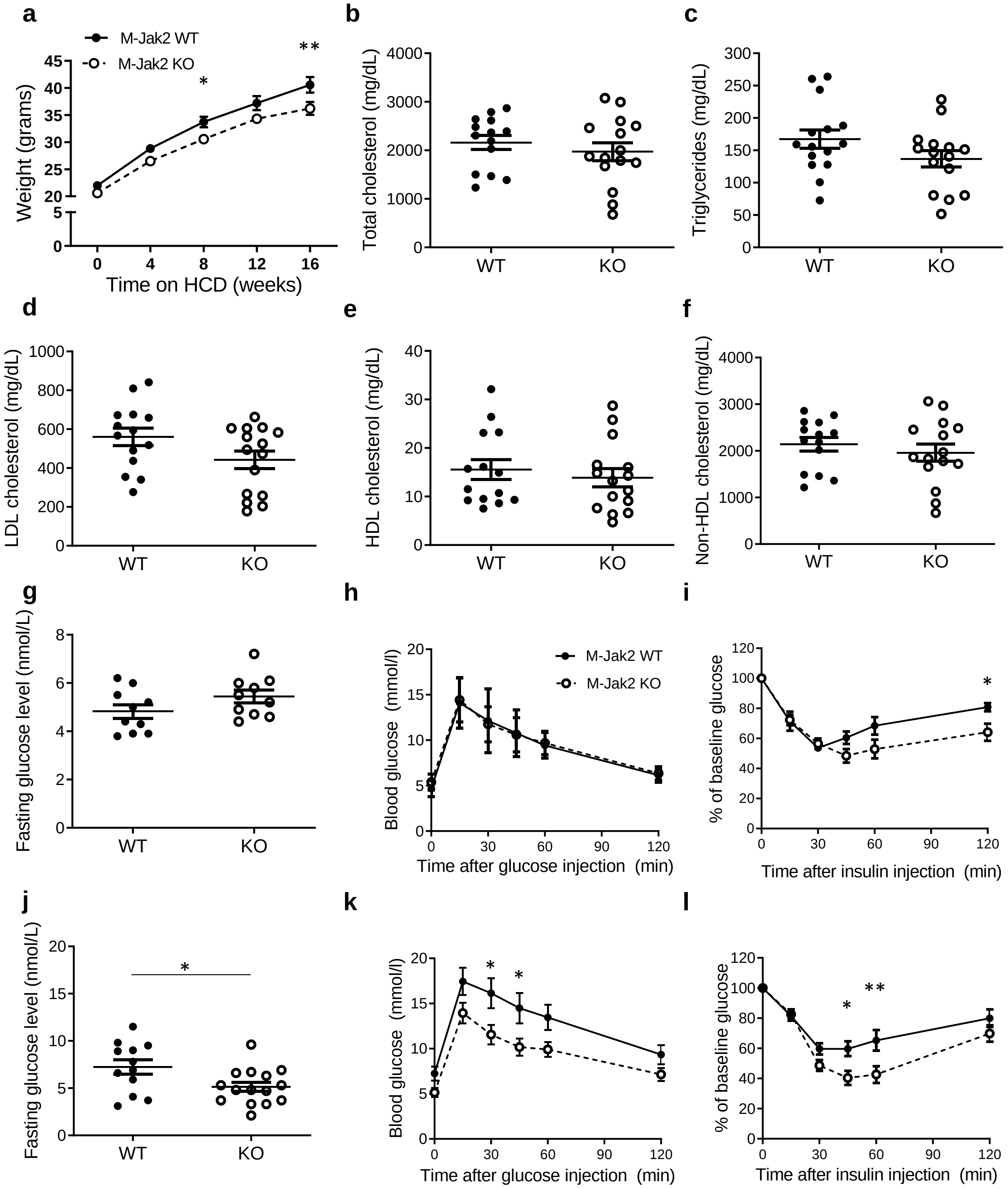

Figure S5. Metabolic parameters in M-Jak2 KO and WT mice. (a) Measurements of body weight while on 16 weeks of HCD (n=25-37/genotype), **(b-f)** their lipid profiles (n=14-15/genotype), **(g-i)** their fasting blood glucose, GTT, and ITT at baseline before starting HCD (n=10-12/genotype), **(j-l)** and after 16 weeks of HCD (n=12-15/genotype). Statistical analysis: (a, h-i, k-l) Multiple t-tests with Holm-Sidak correction and (b-g, j) two-tailed unpaired t-tests were performed. Data are presented as the mean ± SEM, *p<0.05, **p<0.01.

Figure S6

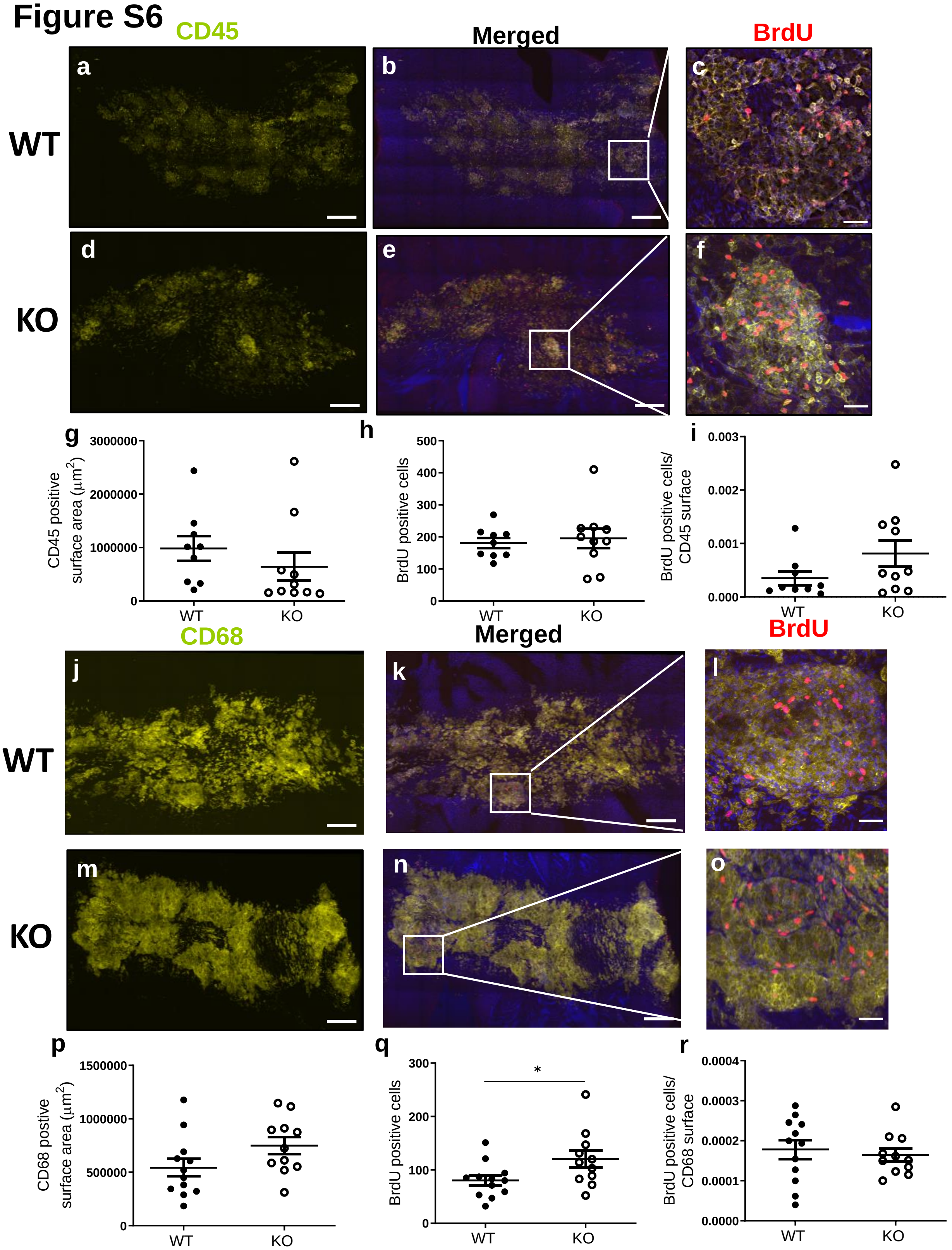

Figure S6. Macrophage recruitment and proliferation in the lesser curvature of the aortic arch. **(a-i)** 24-hour BrdU incorporation assessment in M-Jak2 KO and WT mice fed 16 weeks of HCD; **(a and d)** Representative *en face* images immunostained with anti-CD45 antibody 24 hours after BrdU injection. Scale bar, 200 μm. **(b, c, e and f)** Merged images of CD45 and BrdU in the lesser curvature. Scale bar, (b, e) 200 μm; (c, f) 50 μm. **(g and h)** Quantification of CD45 positive surface area (μm^2) and BrdU positive cells, (n=9-10/genotype). **(i)** Normalization of BrdU positive cells to CD45 surface area (n=9-10/group). **(j-r)** 3-hour BrdU incorporation assessment in M-Jak2 KO and WT mice fed 16 weeks of HCD; **(j and m)** Representative images of the lesser curvature of the aorta immunostained with anti-CD68 3 hours after BrdU injection. Scale bar, 200 μm. **(k, l, n and o)** Merged images of BrdU in the lesser curvature. Scale bar, (K, N) 200 μm; (L, O) 50 μm. **(p and q)** Quantification of CD68 positive surface area (μm^2) and BrdU positive cells (n=11-12/genotype). **(r)** Normalization of BrdU positive cells to CD68 surface area (n=11-12/genotype). Statistical analysis: Two-tailed unpaired t-tests were performed. Data are presented as the mean ± SEM, *p<0.05.

Figure S7

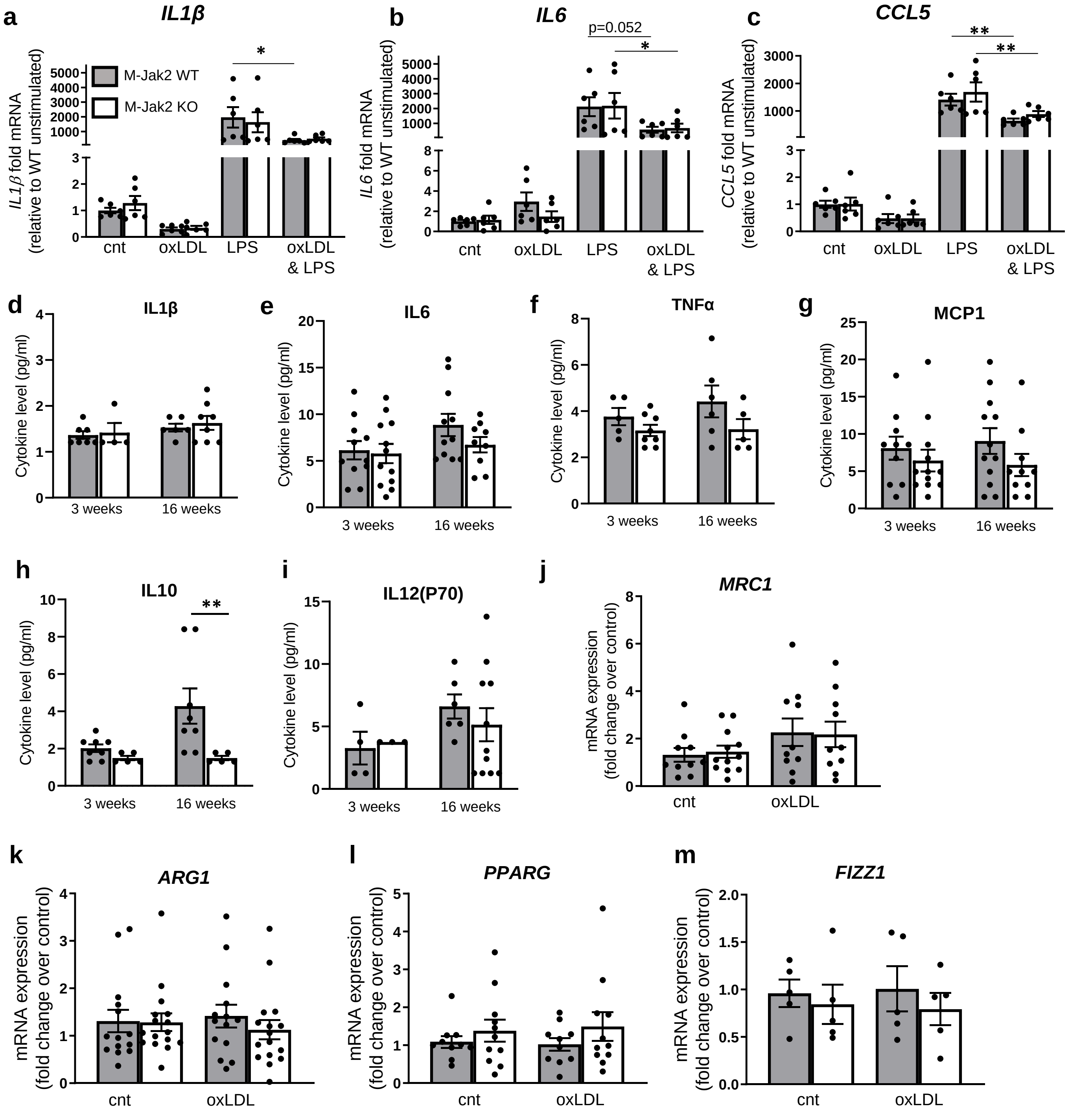

Figure S7. Gene expression of markers of inflammation in BMDM and serum cytokines of M-Jak2 KO and WT mice. (a-c) mRNA levels of *IL1β* **(a)**, *IL6* **(b)** and *CCL5* **(c)** from BMDM loaded with oxidized LDL (OxLDL) (100 µg/ml for 24h) and subsequently stimulated with LPS (10 ng/ml) for 6 hours (n=6/group) from 6-8 week old mice. **(d-i)** Serum cytokines (IL-1β: n=4-8/group; IL6: n=9-12/group; TNFα: n=5-8/group; MCP1: n=10-12/group; IL10: n=5-8/group; IL12(P70): n=3-11/group) after 3 weeks and 16 weeks of HCD. **(j-m)** mRNA level of *MRC1* (n=10-12/group), *ARG1* (n=14-16/group), *PPARG* (n=10-11/group) and *FIZZ1* (n=5/group) from BMDM of M-Jak2 KO and WT mice stimulated with oxLDL for 24 hours. Statistical analysis: (a-c) Two-way ANOVA with Tukey's multiple comparisons, (d-i) unpaired t-tests at 3 and 16 weeks and (j-m) multiple t-tests with Holm-Sidak correction. Data are presented as the mean ± SEM, *p<0.05, **p<0.01.

Figure S8

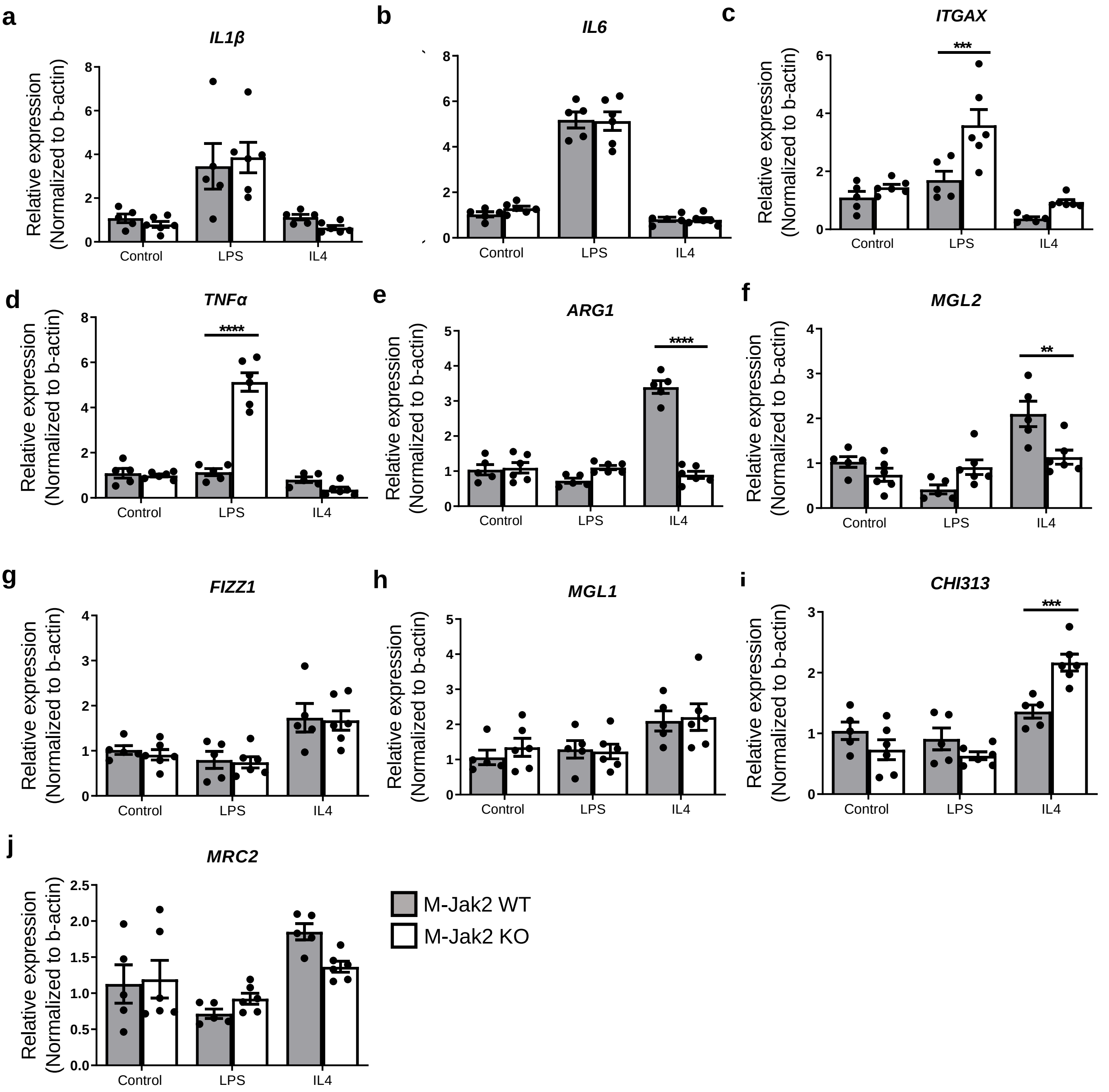

Figure S8. Polarization of BMDM from M-Jak2 KO and WT mice. mRNA expression of **(a-d)** M1 and **(e-j)** M2 markers in BMDM from M-Jak2 KO mice and WT control mice in response to LPS and IL4 for 24 hours. Values are normalized to β-actin mRNA levels and presented as fold change over untreated WT BMDM (n=5-6/group). Statistical analysis: Two-way ANOVA with Tukey's multiple comparisons. Data are presented as the mean ± SEM, $^{**}p < 0.01$, $^{***}p < 0.001$, $^{****}p < 0.0001$.

Figure S9

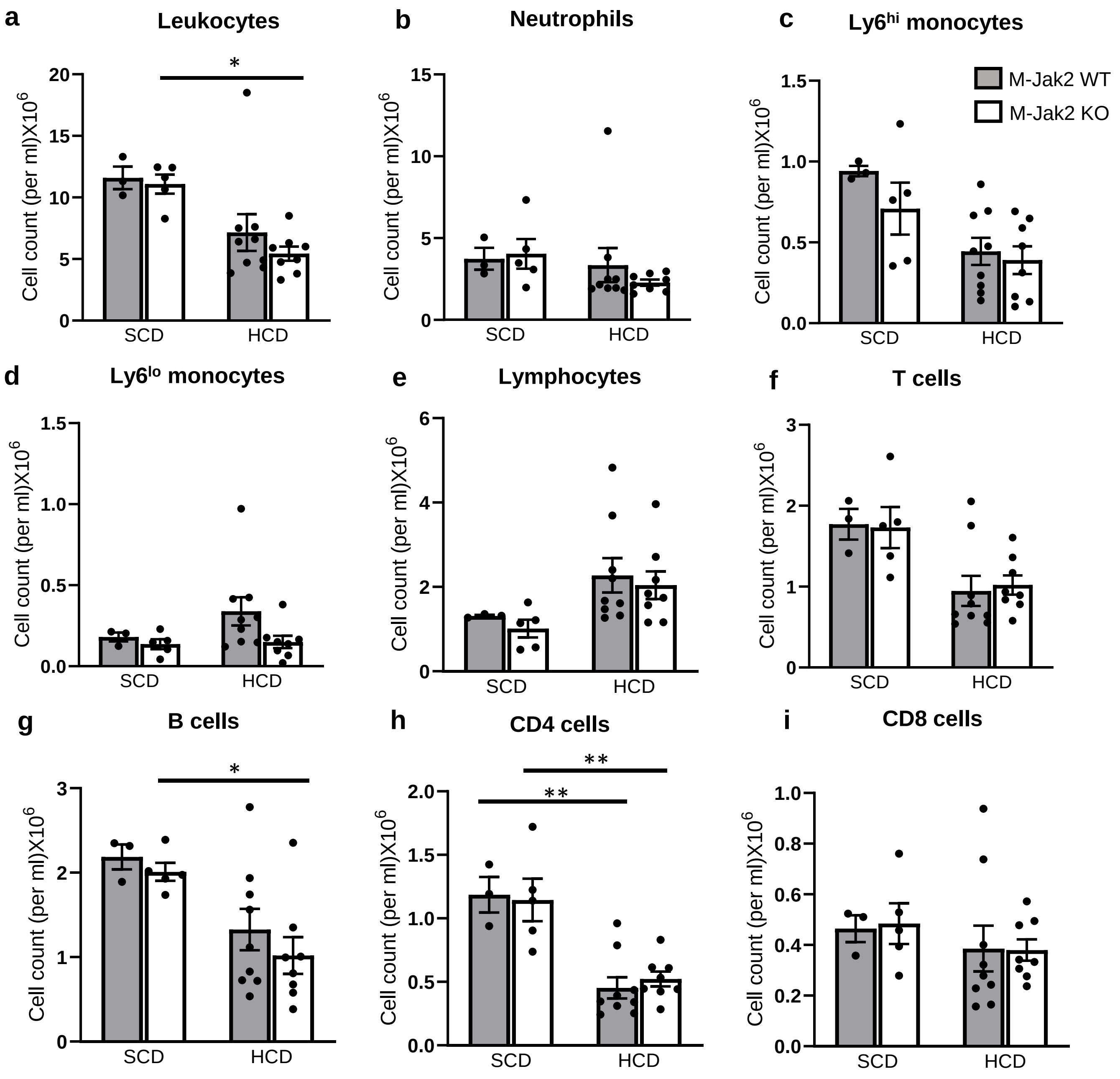

Figure S9. Circulating leukocyte measurements of M-Jak2 KO and WT mice. (a-i) Leukocyte counts by flow cytometry from peripheral blood of M-Jak2 KO and WT control mice at age of 6 weeks (fed with SCD) as well as after 16 weeks of HCD (n=3-9/group). Statistical analysis: Two-way ANOVA with Tukey's multiple comparisons was performed. Data are presented as the mean ± SEM. *$p<0.5$, **$p < 0.01$.

Figure S10

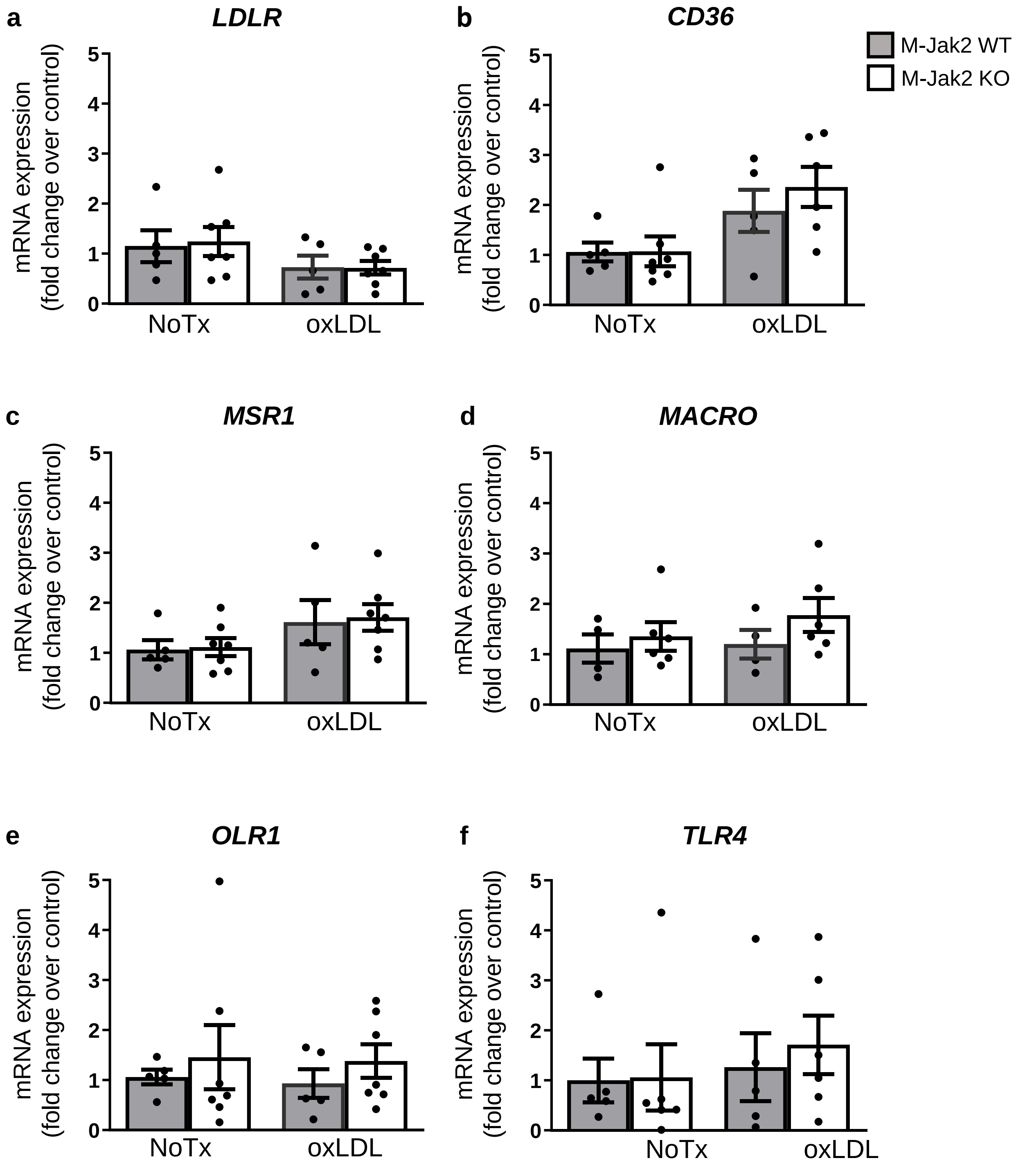

Figure S10. Gene expression of cholesterol uptake receptors. (a-f) mRNA level of *LDLR* **(a)**, *CD36* **(b)**, *MSR1* **(c)**, *MARCO* **(d)**, *OLR1* **(e)**, and *TLR4* **(f)** in BMDM of M-Jak2 KO and WT mice at baseline and after 24 hours with oxLDL (n=5-7/group). Statistical analysis: Multiple t-tests with Holm-Sidak correction were performed. Data are presented as the mean ± SEM.

Figure S11

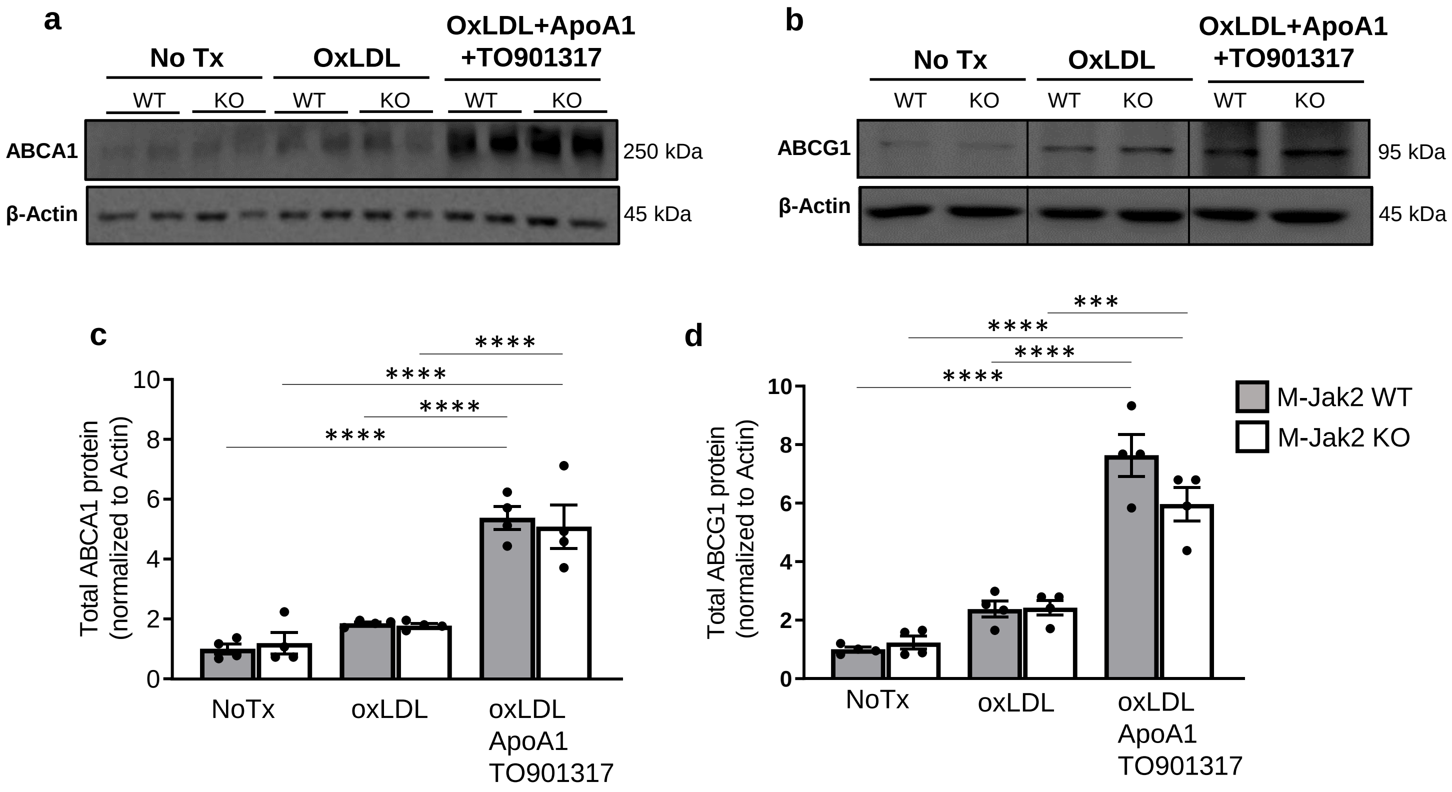

Figure S11. ABCA1 and ABCG1 protein levels in response to oxLDL, TO901317 and ApoA1. (a and b) Representative western blots of ABCA1 (a) and ABCG1 (b) in BMDM treated with OxLDL alone or combined with TO901317 and ApoA1, **(c and d)** and their quantification, normalized to β-actin (n=4/group). The lanes in panel B were run on the same gel but were noncontiguous. Statistical analysis: Two-way ANOVA with Tukey's multiple comparisons was performed. Data are presented as the mean ± SEM, ***p<0.001, ****p<0.0001.

Figure S12

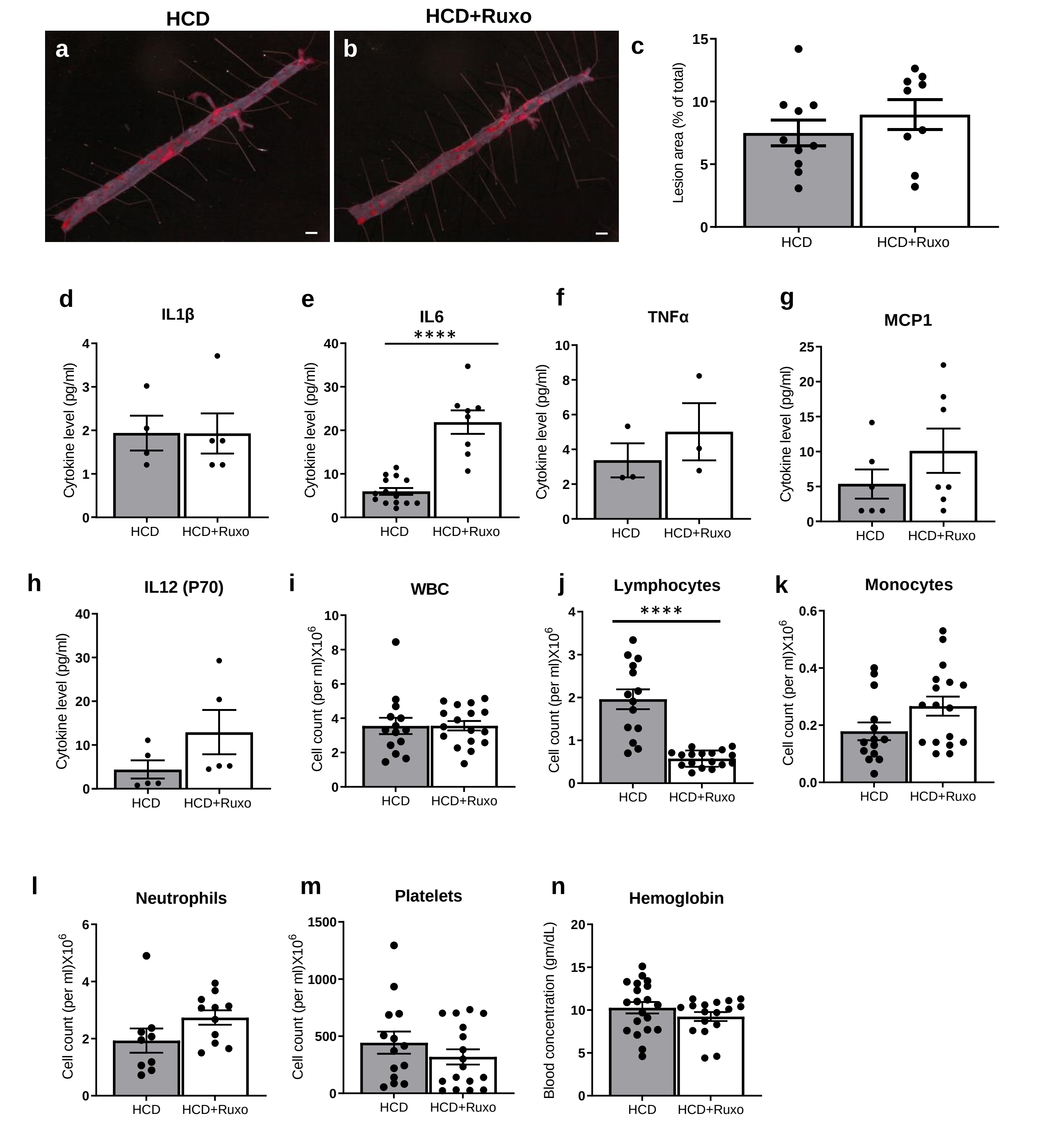

Figure S12. Effects of ruxolitinib in atherosclerosis of descending aorta, leukocyte panel and serum cytokines. (a and b) Representative images of *en face* Oil-red-O staining of atherosclerotic plaque area in the descending aorta in ApoE KO mice fed either HCD with or without ruxolitinib for 16 weeks. Scale bar, 200 μm. **(c)** Quantification of lesion size at the descending aorta (μm^2) (n=9-10/genotype). **(d-h)** Serum cytokines (IL-1β: n=4-5/genotype; IL6: n=8-14/genotype; TNFα: n=3/genotype; MCP1: n=6-7/genotype; IL12(P70): n=5/genotype) of ApoE KO mice fed either HCD with or without ruxolitinib for 16 weeks. **(i-n)** Leukocyte counts by flow cytometry from peripheral blood of ApoE KO mice fed either HCD with or without ruxolitinib for 16 weeks (n=14-17/genotype). Statistical analysis: Two-tailed unpaired t-tests were performed. Data are presented as the mean ± SEM, ****$p<0.0001$.

Figure S13

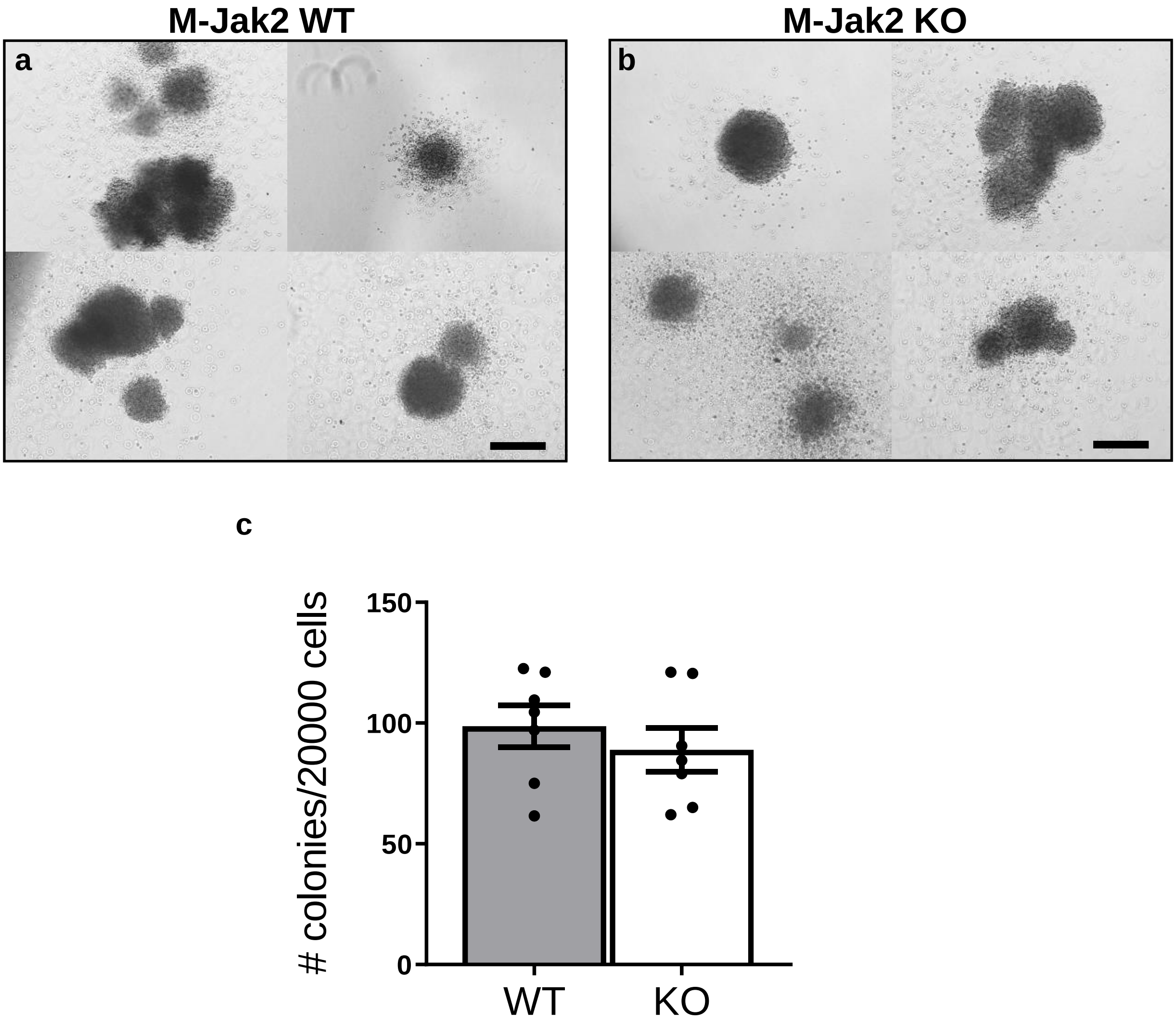

Figure S13. Clonogenic growth assay in BM cells of M-Jak WT and KO mice. (a and b) Representative images showing clonogenic growth assay on freshly collected BM cells from M-Jak2 WT and KO mice. Images are at 20X magnification. Scale bars, 1 mm. **(c)** Quantification of GM-CFU per 20000 seeded cells (n=7/genotype). Statistical analysis: Two-tailed unpaired t-test was performed. Data are presented as the mean ± SEM.

Figure S14

Source data for Fig. S1 & S11- unprocessed western blot

S1c

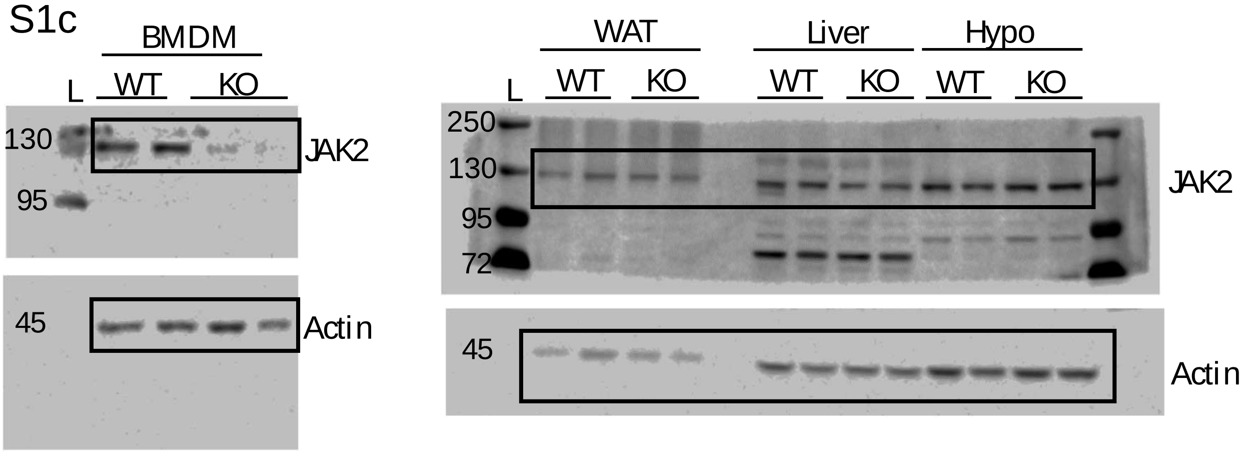

S11a

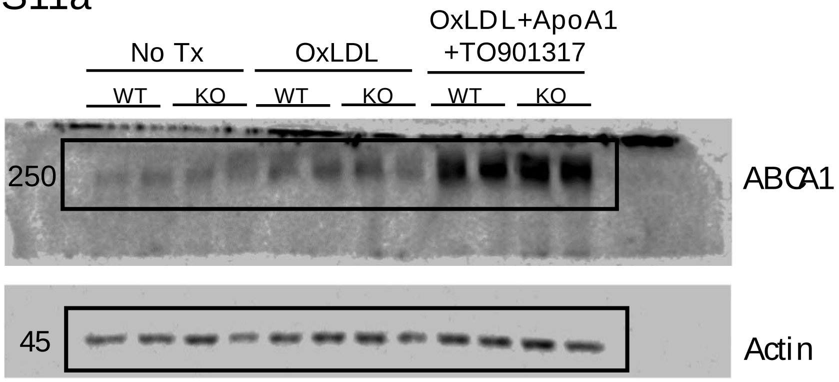

S11b

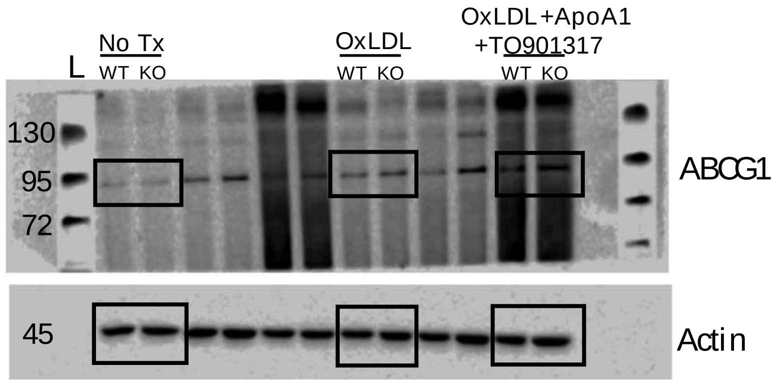

Supplementary Tables

Table S1: Quantitative RT-PCR primers sequences

Gene	Sequence
ABCA1	F: 5’-AAA ACC GCA GAC ATC CTT CAG-3’
	R: 5’-CAT ACC GAA ACT CGT TCA CCC-3’
ABCG1	F: 5’-ATC TGA GGG ATC TGG GTC TGA-3’
	R: 5’-CCT GAT GCC ACT TCC ATG A-3’
SCARB1	F: 5’-TCC CTT CGT GCA TTT TCT CA-3’
	R: 5’-GTT CAT CCC AAC AAA CAG GC-3’
MRC1	F: 5’-ATC CAC TCT ACT CAC CTT CA-3’
	R: 5’-TGC TTG TTC ATA TCT GTC TTC A-3’
ARG1	F: 5’-CTC CAA GCC AAA GTC CTT AGA G-3’
	R: 5’-AGG AGC TGT CAT TAG GGA CAT C-3’
PPARG	F: 5’-ACC ACT CGC ATT CCT TTG AC-3’
	R: 5’-AAG GCA CTT CTG AAA CCG AC-3’
IL1β	F: 5’-AGTTGACGGACCCCAAAAGA-3’
	R: 5’-TGCTGCTGCGAGATTTGAAG-3’
IL6	F: 5’-CTCCCAACAGACCTGTCTATACCA-3’
	R: 5’-TGCCATTGCACAACTCTTTTCT -3’
ITGAX	F: 5’-CTG GAT AGC CTT TCT TCT GCT G-3’
	R: 5’-GCA CAC TGT GTC CGA ACT CA-3’
TNFα	F: 5’-CTG TGAAGGGAATGGGTGTT-3’
	R: 5’-TTG GAC CCT GAG CCA TAA TC-3’
MGL2	F: 5’-CAA TGT GCT TAG CTG GAT GGG-3’
	R: 5’-CCA TGC CAG TTA TCC GGC TG-3’
FIZZ1	F: 5’-CCA ATC CAG CTA ACT ATC CCT CC-3’
	R: 5’-ACC CAG TAG CAG TCA TCC CA-3’
MGL1	F:5’-CAC CAT GAT ATA CGA AAA CCT CCA GAA CTC-3’

	R:5’-CTA GCT CTC CTT GGC CAG C-3’
CHI313	F:5’-CAG GTC TGG CAA TTC TTC TGA A-3’
	R:5’-GTC TTG CTC ATG TGT GTA AGT GA-3’
MRC2	F: 5’-TCT CCC GGA ACC GAC TCT TC-3’
	R: 5’-AAC TGG TCC CCT AGT GTA CGA-3’
CCL5	F: 5’-CCTGCTGCTTTGCCTACCTCTC-3’
	R: 5’-ACACACTTGGCGGTTCCTTCGA-3’
LDLR	F: 5’-CAG CTC TTC ACC GGA GAC C-3’
	R: 5’-ACC TGC TGT GTC CTA GCT GG-3’
CD36	F: 5’-GCC AAG CTA TTG CGA CAT GA-3’
	R: 5’-AAA AGA ATC TCA ATG TCC GAG ACT TT-3’
MSR1	F: 5’-CTG GAC AAA CTG GTC CAC CT-3’
	R: 5’-TCC CCT TCT CTC CCT TTT GT-3’
MACRO	F: 5’-CCA CCT GAT CCT GCT CAC GGC-3’
	R: 5’-GCC CAC TGC AGC GAG AAG G-3’
OLR1	F: 5’-CTG GAT TGG ATT GCA TCG GAA-3’
	R: 5’-CAG CTC CGT CTT GAA GGT ATG-3’
TLR4	F: 5’-GCA ATG TCT CTG GCA GGT GTA-3’
	R: 5’-CAA GGG ATA AGA ACG CTG AGA-3’

Table S2: Information of MNP patients with *Jak2*^{V617F} mutation

Sample ID	Gender	Age at Diagnosis
80367	Female	76
120012	Female	60
120072	Female	58
130084	Female	48
150424	Female	60
150690	Female	72
151295	Female	30
186465	Female	47
213440	Female	61
304707	Female	43
311833	Female	61
90624	Male	65
120501	Male	62
130330	Male	62
130681	Male	62
130909	Male	84
140672	Male	73
140799	Male	66
140973	Male	69
150927	Male	56
329719	Male	62
