# Supplementary material for: Macrophage Jak2 deficiency accelerates atherosclerosis through defects in cholesterol efflux
Source: Commun Biol. 2022 Feb 15;5:132. doi: 10.1038/s42003-022-03078-5 (PMC8847578; doi:10.1038/s42003-022-03078-5)
Supplement: Supplementary file 4 — Reporting Summary [file 42003_2022_3078_MOESM4_ESM.pdf]

nature portfolio

Corresponding author(s): Minna Woo

Last updated by author(s): Dec 21, 2021

Reporting Summary

Nature Portfolio wishes to improve the reproducibility of the work that we publish. This form provides structure for consistency and transparency in reporting. For further information on Nature Portfolio policies, see our Editorial Policies and the Editorial Policy Checklist.

Statistics

For all statistical analyses, confirm that the following items are present in the figure legend, table legend, main text, or Methods section.

n/a	Confirmed	
☐	☒	The exact sample size (*n*) for each experimental group/condition, given as a discrete number and unit of measurement
☐	☒	A statement on whether measurements were taken from distinct samples or whether the same sample was measured repeatedly
☐	☒	The statistical test(s) used AND whether they are one- or two-sided<br>*Only common tests should be described solely by name; describe more complex techniques in the Methods section.*
☒	☐	A description of all covariates tested
☐	☒	A description of any assumptions or corrections, such as tests of normality and adjustment for multiple comparisons
☐	☒	A full description of the statistical parameters including central tendency (e.g. means) or other basic estimates (e.g. regression coefficient) AND variation (e.g. standard deviation) or associated estimates of uncertainty (e.g. confidence intervals)
☐	☒	For null hypothesis testing, the test statistic (e.g. *F*, *t*, *r*) with confidence intervals, effect sizes, degrees of freedom and *P* value noted<br>*Give P values as exact values whenever suitable.*
☒	☐	For Bayesian analysis, information on the choice of priors and Markov chain Monte Carlo settings
☒	☐	For hierarchical and complex designs, identification of the appropriate level for tests and full reporting of outcomes
☒	☐	Estimates of effect sizes (e.g. Cohen's *d*, Pearson's *r*), indicating how they were calculated

Our web collection on statistics for biologists contains articles on many of the points above.

Software and code

Policy information about availability of computer code

Data collection	Flow cytometry data were collected using BD Fortessa or LSRII flow cytometer; confocal images were acquired using Yokogawa CSU10 spinning disk system. Images were acquired using a ZEISS 63x/1.4 NA oil objectives or 25x/0.8 NA water objective and additional 1.5x magnifying lens; real-time PCR results were collected using Applied Biosystems 7900 HT Fast-Real-Time PCR System; weestern blot images were collected by using DNR Bio-Imaging Systems MicroChemi 4.2; radioactivity was counted using PerkinElmer Tri-Carb 2800TR Liquid Scintillation Analyzer.
Data analysis	Statistical analyses were performed with Graphpad Prism 8.3; flow cytometry data analysis using FlowJo V10.6.1 (Treestar); confocal images were processed and analysed using MetaMorph software; images were quantified and analyzed by ImageJ; Routine analyses were done using Volocity software; qPCR data were analyzed by QuantStudio Real-Time PCR Systems.

For manuscripts utilizing custom algorithms or software that are central to the research but not yet described in published literature, software must be made available to editors and reviewers. We strongly encourage code deposition in a community repository (e.g. GitHub). See the Nature Portfolio guidelines for submitting code & software for further information.

Data

Policy information about availability of data

All manuscripts must include a data availability statement. This statement should provide the following information, where applicable:

- Accession codes, unique identifiers, or web links for publicly available datasets
- A description of any restrictions on data availability
- For clinical datasets or third party data, please ensure that the statement adheres to our policy

The authors declare that all data supporting the findings of this study are available within the paper and its supplementary information files.

Field-specific reporting

Please select the one below that is the best fit for your research. If you are not sure, read the appropriate sections before making your selection.

☒ Life sciences ☐ Behavioural & social sciences ☐ Ecological, evolutionary & environmental sciences

For a reference copy of the document with all sections, see nature.com/documents/nr-reporting-summary-flat.pdf

Life sciences study design

All studies must disclose on these points even when the disclosure is negative.

Sample size	Samples sizes were predetermined based on our experience with experiments which is based on statistical power calculations. For experiments with a high variability, we used n>10. For assays with a low variability, we used n<10.
Data exclusions	No data were excluded
Replication	The findings were reproduced in multiple independent experiments. The number of independent experiments and samples for each data panel is indicated in the figure legend. Data in the figures represent the incorporation of all independent experiments. Images included in figures are from corresponding representative experiments.
Randomization	No randomization methods were performed for this study. Experimental samples were allocated randomly to experiments and processed in an arbitrary order.
Blinding	All data were collected in a manner blinded to sample identify. After analyzed the data, the samples were re-identified to interpret the results.

Reporting for specific materials, systems and methods

We require information from authors about some types of materials, experimental systems and methods used in many studies. Here, indicate whether each material, system or method listed is relevant to your study. If you are not sure if a list item applies to your research, read the appropriate section before selecting a response.

Materials & experimental systems

n/a	Involved in the study	
☐	☒	Antibodies
☒	☐	Eukaryotic cell lines
☒	☐	Palaeontology and archaeology
☐	☒	Animals and other organisms
☐	☒	Human research participants
☒	☐	Clinical data
☒	☐	Dual use research of concern

Methods

n/a	Involved in the study	
☒	☐	ChIP-seq
☐	☒	Flow cytometry
☒	☐	MRI-based neuroimaging

Antibodies

Antibodies used

The following antibodies were used in this study:
Alexa Fluor® 647 anti-CD68 antibody (clone FA-11), BioLegend, cat. # 137003 Immunofluorescence
Alexa Fluor® 647 anti-CD45 antibody (clone 30-F11), BioLegend, cat. # 103123 Immunofluorescence
biotin-conjugated anti-BrdU (clone MoBu-1), BioLegend, cat. # 317904 , immunofluorescence
BV650 anti-CD11b (clone M1/70), BD Pharmigen, cat: # 563402, flow cytometry
FITC anti-Mouse CD3e (clone 145-2C11), BD Pharmigen, cat: 553061, flow cytometry
PE-Cy™7 Rat Anti-Mouse CD45 (clone 30-F11), BD Pharmigen, cat: no:561868, flow cytometry
PE Anti-Mouse Ly-6G (clone 1A8), BD Pharmingen, cat: # 551461, flow cytometry.
FITC anti–mouse Ly-6C (clone AL-21), BD Pharmingen, cat: # 561085, flow cytometry
APC anti–CD115-APC (clone AFS98), eBioscience, cat: # 17-1152-82, flow cytometry
Jak2 monoclonal rabbit antibody (Clone D2E12), Cell Signaling Technology, cat: #3230, western blot
ABCA1 polyclonal rabbit antibody, Novus Biologicals, cat: # NB400-105, western blot
SR-B1 polyclonal rabbit antibody, Novus Biologicals, cat: #NB400-104, western blot
ABCG1 polyclonal rabbit antibody, Novus Biologicals, cat: #NB400-132, western blot
β-actin rabbit antibody, Cell Signaling Technology, cat: #4967, western blot
HRP-conjugated anti-rabbit IgG, secondary antibody, Cell Signaling Technology, cat: #7074 western blot

Validation

All antibodies are commercially available. Antibody profiles are listed in following web links:
Alexa Fluor® 647 anti-CD68 antibody https://www.biolegend.com/fr-ch/products/alexa-fluor-647-anti-mouse-cd68-antibody-6422
Alexa Fluor® 647 anti-CD45 antibody https://www.biolegend.com/fr-fr/products/alexa-fluor-647-anti-mouse-cd45-antibody-3101
biotin-conjugated anti-BrdU https://www.biolegend.com/en-us/products/biotin-anti-brdu-antibody-3697

BV650 anti-CD11b https://www.bdbiosciences.com/en-ca/products/reagents/flow-cytometry-reagents/research-reagents/singlecolor-
antibodies-ruo/bv650-rat-anti-cd11b.563402
FITC anti-Mouse CD3e https://www.bdbiosciences.com/en-ca/products/reagents/flow-cytometry-reagents/research-reagents/single-color-antibodies-ruo/fitc-hamster-anti-mouse-cd3e.553061
PE-Cy™7 Rat Anti-Mouse CD45 https://www.bdbiosciences.com/en-us/products/reagents/flow-cytometry-reagents/researchreagents/
single-color-antibodies-ruo/pe-cy-7-rat-anti-mouse-cd45.561868
PE Anti-Mouse Ly-6G https://www.bdbiosciences.com/en-us/products/reagents/flow-cytometry-reagents/research-reagents/singlecolor-
antibodies-ruo/pe-rat-anti-mouse-ly-6g.551461
FITC anti–mouse Ly-6C https://www.bdbiosciences.com/en-us/products/reagents/flow-cytometry-reagents/research-reagents/single-color-antibodies-ruo/fitc-rat-anti-mouse-ly-6c.561085
APC anti–CD115-APC https://www.thermofisher.com/antibody/product/CD115-c-fms-Antibody-clone-AFS98-Monoclonal/17-1152-82
Jak2 monoclonal rabbit antibody https://www.cellsignal.com/products/primary-antibodies/jak2-d2e12-xp-rabbit-mab/3230
ABCA1 polyclonal rabbit antibody https://www.novusbio.com/products/abca1-antibody_nb400-105
SR-B1 polyclonal rabbit antibody https://www.novusbio.com/products/sr-bi-antibody_nb400-104h
ABCG1 polyclonal rabbit antibody https://www.novusbio.com/products/abcg1-antibody_nb400-132b
β-actin rabbit antibody https://www.cellsignal.com/products/primary-antibodies/b-actin-antibody/4967
HRP-conjugated anti-rabbit IgG https://www.cellsignal.com/products/secondary-antibodies/anti-rabbit-igg-hrp-linked-antibody/7074

Animals and other organisms

Policy information about studies involving animals; ARRIVE guidelines recommended for reporting animal research

Laboratory animals	Six-week to 22-week-old mice on a C57BL/6 background were used. Both male and female mice were used.
Wild animals	No wild animals were used.
Field-collected samples	No field-collected sample were used.
Ethics oversight	All animal experimental protocols were approved and performed in accordance with the guidelines of the Canadian Council on Animal Care and regulations established by the Toronto General Hospital Research Institute Animal Care Committee (AUP 2862).

Note that full information on the approval of the study protocol must also be provided in the manuscript.

Human research participants

Policy information about studies involving human research participants

Population characteristics	Healthy adult and patients with myeloproliferative neoplasms (PMN) harbouring the JAK2V617F mutation: age 43-84; mixed genders; participants information is listed in supplementary table S1.
Recruitment	Participants samples were recruited by the Elizabeth and Tony Comper MPN Program and the Leukemia Tissue Bank at Princess Margaret Cancer Centre/University Health Network.
Ethics oversight	The research protocol was reviewed and approved by the University Health Network Research Ethics Board (01-0573).

Note that full information on the approval of the study protocol must also be provided in the manuscript.

Flow Cytometry

Plots

Confirm that:

☒ The axis labels state the marker and fluorochrome used (e.g. CD4-FITC).

☒ The axis scales are clearly visible. Include numbers along axes only for bottom left plot of group (a 'group' is an analysis of identical markers).

☒ All plots are contour plots with outliers or pseudocolor plots.

☒ A numerical value for number of cells or percentage (with statistics) is provided.

Methodology

Sample preparation	Peripheral blood was collected from mice into heparin-coated capillary tubes and RBCs were lysed with RBC lysis buffer. Cells were resuspended in FACS buffer and allowed to block non-specific binding in Fc receptor blocking solution.
Instrument	BD Fortessa or LSRII flow cytometer
Software	FlowJo V10.6.1
Cell population abundance	The abundance of the relevant cell populations within the single cell population was 90-100%

Gating strategy	FSC/SSC gating of the starting cell population, gating on circulating immune cell distribution as previously described (Yu YR et al. 2016; Plos One)

☐ Tick this box to confirm that a figure exemplifying the gating strategy is provided in the Supplementary Information.
